# Neuroprotective strategies in multiple sclerosis: a status update and emerging paradigms

**DOI:** 10.1080/14737175.2025.2510405

**Published:** 2025-06-03

**Authors:** Catalina I. Coclitu, Cris S. Constantinescu, Radu Tanasescu

**Affiliations:** a Department of Multiple Sclerosis and Neuroimmunology, CHU Grenoble, Grenoble, France; b Cooper Neurological Institute, Cooper Medical School of Rowan University, Camden, NJ, USA; c Academic Unit of Mental Health and Clinical Neurosciences, Clinical Neurology, University of Nottingham, Nottingham, UK; d Department of Neurology, Nottingham Centre for MS and Neuroinflammation, Nottingham University Hospitals NHS Trust, Nottingham, UK

**Keywords:** Multiple sclerosis, neurodegeneration, neuroprotection, demyelination, axonal regeneration

## Abstract

**Introduction:**

MS is a disease continuum in which maladaptive inflammation and neurodegeneration co-occur from onset and evolve over time. Recent progress in the understating of MS pathobiology creates new perspectives for novel neuroprotective therapeutic strategies.

**Areas covered:**

The authors briefly review the mechanisms underlying inflammation and neurodegeneration in MS and discuss the current and emerging strategies to promote neuroprotection in MS. Data were derived in large part from extensive review of the published literature available on PubMed (up to 5th of March 2025).

**Expert opinion:**

Strategies for neuroprotection should be ideally implemented early in the course of MS. They should consider the interplay between neuroinflammation, demyelination and neurodegeneration, the maladaptive changes in the CNS innate immunity resident cells, axonal mitochondrial dysfunction (axonal response of mitochondria to demyelination, ARMD), and remyelination. There is a need for adequate biomarkers that can help to monitor outcomes of target engagement. Comorbidities and aging can worsen neurodegeneration and impair neuroprotective/regenerative processes. Candidate drugs from preclinical and early clinical studies should be tested in multi-arm multistage adaptive trials.

## 1. Introduction

Multiple sclerosis (MS) is a chronic and disabling immune-mediated neurological disorder associated with damage to the central nervous system (CNS) through inflammation, demyelination, and neuronal degeneration [1]. MS age of onset spans from childhood to adult life. Most people with MS experience the first clinical demyelinating episode between 20 and 30 years of age, however in recent years the onset of MS shows a shift toward an older age [2].

The classical descriptive clinical concept of MS refers traditionally to clinical phenotypes (relapsing-remitting, secondary progressive and primary progressive) with or without inflammatory activity [3]. However, the current understanding of MS is of a continuum of pathophysiological events rather than distinct categories, highlighting significant interindividual variability in disease presentation and progression [4].

The etiology of MS is multifactorial, with genetic predisposition accounting for approximately 30% of the risk, while environmental and epigenetic factors contribute significantly to disease susceptibility [5,6]. Studies indicate that the interaction between genetic factors and environmental influences, such as vitamin D deficiency and microbial exposure, plays a crucial role in MS pathogenesis [7,8]. Furthermore, epigenetic modifications, particularly DNA methylation [9], have been implicated in the development and progression of the disease, suggesting that these alterations may serve as potential therapeutic targets [10] For example, aberrant DNA methylation profiles have been associated with inflammation and neurodegeneration in MS patients, indicating a complex interplay between genetic and epigenetic factors [6,11].

Tissue damage in MS results from a complex interplay between the peripheral immune system, glia and neuron-specific factors [12]. Multifocal CNS inflammation that is periphery-driven is found in early MS pathology; as the disease progresses, the adaptive immune system outside the CNS loses its cardinal role and CNS specific immunity with the activation of microglia and astroglia leading to demyelination, neuroaxonal degeneration, oligodendroglial death, and astrocytic scarring [12]. Pathology data show that inflammation drives tissue injury at all stages of MS [13]. MS lesions can be seen as focal areas of demyelination due to perivenular inflammation and glial reaction, with the involvement of the innate and the adaptive immune system [13]. The focal inflammatory meningeal and perivascular infiltrates produce soluble factors that contribute to demyelination and neurodegeneration and can induce microglia activation [13]. In addition, oxidative injury and mitochondrial damage contribute to demyelination and neurodegeneration [13]. Biopsies of initial active lesions identified three main particular immunological patterns of demyelination which are well described, with an essential role of T cell infiltration [13]. The processes that follow the initial acute inflammation are not well understood [14]. In approximately 20% of the lesions, the inflammatory process results in chronic organized damage with infiltrates of tissue-resident CD8+ memory cells, monocytes and activation of astrocytes and microglia [4].

Gray matter neurodegeneration is considered to have a pivotal role in MS disease progression [15]. Gray matter MS lesions form subpial lesions that can be extensive and can be associated with meningeal inflammation [16]. Leptomeninges play an important role in chronic inflammation, with a correspondence between leptomeningeal inflammation and demyelination in the subpial cortex [16]. Cortical lesions seem to be less inflammatory when captured in biopsies made after several years of disease. Nevertheless, within this low-grade inflammation in the gray matter brain tissue (not only cortical lesions), the blood-brain barrier (BBB) is damaged and there is a high density of ramified microglia, astrogliosis, and deposition of complement proteins [16].

**Figure 1.**
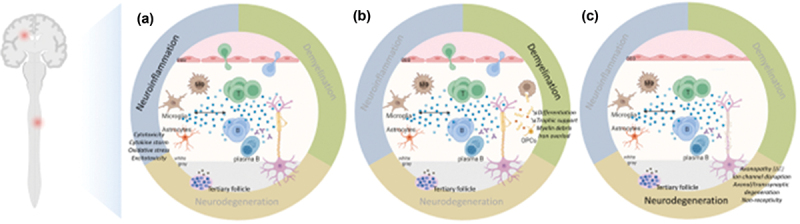
The pathogenesis of multiple sclerosis (figure and legend reproduced from [18]).

Demyelination, oligodendroglial loss, and axonal pathology are the main contributors to neurodegeneration. Molecular pathways involved in axonal damage and neurodegeneration include oxidative stress, calcium overflow, excitotoxicity, and mitochondrial damage [17,18] (Figure 1, Supplementary Table S1). Strategies that aim to promote neuroprotection should be able to reverse and repair the mechanisms along these pathways [19]. The development of MS toward a progressive course signals a partial shift from a predominantly localized acute disease to extensive inflammation and neurodegeneration coinciding with the failure of remyelination and of neuroplasticity [4]. However, classifying MS with accurate clinical correspondents based on biological mechanisms is challenging [20]. The terms ‘progression independent of relapse activity’ (PIRA) [21] and ‘silent progression’ [22] refer to accumulation of disability in relapsing MS which occurs separately and independently of the relapse-associated worsening (RAW). PIRA often goes undetected due to the limitations of follow-up assessments, but is present even in the early stages of MS [23]. The concept of smoldering-associated-worsening (SAW), which includes subtle clinical deterioration beyond the definition of PIRA, resulting from smoldering pathological processes, can have diverse possible pathological substrates [24] and immune-metabolic mechanisms [17].

(a) Neuroinflammation is marked by the invasion of peripheral immune cells in the CNS through a disrupted BBB in the early inflammatory phase of MS. These cells are reactivated, secrete cytokines and cytotoxic molecules, attract more peripheral immune cells and activate macrophages, microglia, and astrocytes, which produce cytokines, nitric oxide, and reactive oxygen species (ROS) (blue dots). B cells can also differentiate into autoantibody-producing plasma cells. With disease progression, infiltration of peripheral immune cells is reduced since the BBB is closed. CNS-resident cells, i.e. microglia and astrocytes, sustain the inflammation by producing cytokines (TNFa, IL6) and releasing ROS (blue dots). TNFa-mediated glutamate release from microglia and its impaired turnover by astrocytes result in excitotoxicity. Plasmablasts and plasma B cells form tertiary follicle-like structures in the meninges that may release proinflammatory factors activating microglia (brown dots).

(b) Demyelination is partly caused by this cytotoxic and proinflammatory environment that breaks down the myelin sheaths. Macrophages and microglia, attracted by astrocytes, can clear the myelin debris, allowing (partial) remyelination by surviving oligodendrocytes or by OPCs proliferating, migrating, and differentiating at the site of injury in response to cytokines, chemokines, mitogens (platelet-derived growth factor), chemo-attractants (semaphorin 3F), and trophic factors (insulin-like growth factor, ciliary neurotrophic factor) (blue dots) released by microglia and astrocytes. This will reduce the harm to the axons. However, the phagocytic capacity of microglia/macrophages decreases with disease progression. Hence, myelin debris are improperly cleared, trigger an inflammatory response, and inhibit axonal growth, and OPCs are less recruited and fail to differentiate. The trophic support of oligodendrocytes to the underlying axons wanes. The ferrous iron (red dots) released from the myelin, where it accumulates with age, is oxidized to ferric iron, producing ROS and causing lipid peroxidation and ferroptosis.

(c) Neurodegeneration starts early in the disease and becomes prominent with disease progression when the compensatory mechanisms safeguarding the CNS reserve are exceeded. Axons are directly harmed by the proinflammatory and oxidative environment, but also by the loss of the insulating and supporting myelin sheaths. Chronically demyelinated axons seem to be non-receptive to OPCs. Nodal and paranodal ion channels are disorganized; synapses are dysfunctional. Axons suffer a major energy debt altering axonal transport of mitochondria and synaptic vesicles. The axonopathy spreads the axonal and transsynaptic degeneration. These events are self-sustained and intermingled, further enhanced by senescent processes, resulting in a major cytokine storm and oxidative burst, mitochondrial dysfunction, mitochondrial DNA damage, energy failure, ion imbalance, cytotoxicity, excitotoxicity, lack of trophic support by the loss of oligodendrocytes, and axonal loss.

BBB = blood – brain barrier; B = B cell; CNS = central nervous system; ∆E = energy deficit: MS = multiple sclerosis; OPC = oligodendrocyte progenitor cell; ROS = reactive oxygen species; *T* = T cell; Th = T helper cell.

Despite the advances in understanding MS, halting and reversing MS progression remains a critical challenge. Current treatments in MS are directed against the inflammatory component of MS, while emerging therapeutic strategies often target specific damaging mechanisms in isolation, although it is increasingly recognized that these mechanisms are interconnected [4,25]. The need for a holistic approach that considers the multifaceted nature of MS, including its immunological, genetic, and environmental components, is essential for developing more effective treatment strategies. Neuroprotection refers to an effect that may result in salvage, recovery, or regeneration of the CNS cells, structure, and function [26]. In this review, we explore briefly the mechanisms underlying inflammation and neurodegeneration in MS and the current and emerging strategies to promote neuroprotection in MS. Data was extracted from a medical literature review (pubmed.ncbi.nlm.nih.gov) conducted up to 5^th^ of March 2025, using the keywords ‘neuroprotection, MS, neurodegeneration, molecular mechanisms, microglia, astrocyte, remyelination.’ We first refer to the principles of neuroprotection through preventing peripheral-driven inflammation, and then outline CNS inflammation and mechanisms of neuronal injury including neuronal death, remyelination and axonal regeneration in MS. We further comment on lifestyle factors and neuroprotection in MS, and discus fluid and radiological biomarkers for progression and neurodegeneration.

## 2. Neuroprotection through preventing peripheral-driven inflammation in MS

### 2.1. Disease modifying therapies

The emergence of disease-modifying treatments (DMTs) has revolutionized the treatment of MS, especially of the relapsing forms. The early use of DMTs curtails inflammation by cutting off the migration of the lymphocytes into the CNS, by depleting specific types of immune cells, by interfering with several pathways in lymphocyte maturation, or by blocking their activation and effector mechanisms, essentially having an anti-inflammatory and immunomodulatory role. Having fewer inflammatory episodes in the early disease phases seems to limit the subsequent propensity to neurodegeneration and therefore provide neuroprotection. However, despite having reduced disability progression [27], DMTs do not cure MS. DMTs effects are still limited in primary progressive MS, and despite the fact that longer DMT exposure reduces the risk of converting to SPMS [28], this risk is not eradicated. While the currently approved DMTs are effective in preventing peripheral immune attacks and as a consequence are useful in preventing focal lesion formation and reducing clinical and radiological relapses, they are less successful in modulating smoldering disease activity and preventing PIRA [29,30].

DMTs can be grouped by their mode of action in therapies with pleiotropic effects, therapies reducing immune cell proliferation, therapies depleting immune cells and therapies reducing immune cell migration.

Interferon-beta (IFNβ) and glatiramer acetate (GA) were the first immunomodulatory therapies approved for MS. Their efficacy is mild but well established with mild persistent effect in controlling inflammation. IFNβ reduces the relapse rate by up to 33% [31] and GA reduces the relapse rate by 29% [32]. These therapies have pleiotropic effects. This is also the case for dimethyl fumarate (DMF), another well-established DMT. All three DMTs are effective only in relapsing-remitting forms of MS. DMF has a complex mechanism of action principally targeting the Nrf2 transcription factor involved both in lymphogenesis and in the oxidative stress response [33]. DMF reduces the relapse rate with around 44% in CONFIRM trial when compared to GA [34] and 41% in DEFINE trial [35].

Historically, nonspecific immunosuppressants such as azathioprine, cyclophosphamide, and mitoxantrone were used as treatment for MS, but their use is limited by an unfavorable risk-to-benefit ratio [36].

Teriflunomide is a immunomodulatory pyrimidine synthesis inhibitor with similar efficacy on reducing relapse rate to IFNβ [37], a good safety profile [38], and potential positive effects on slowing down accumulation of gray matter and normal-appearing white matter microstructural damage [39].

Immune reconstitution therapies with a depletion of selected immune cell populations are well-established effective strategies in MS treatment. Alemtuzumab targets CD52, a pan-lymphocyte cell surface molecule, removing almost all lymphocytes from the blood flow, with T lymphocytes being undetectable for 18 months after one cycle of treatment [40]. Relapse rate reduction is 74% and the risk of sustained disability progression was reduced by 71% by alemtuzumab when compared to IFNβ [41,42].

Another immune reconstitution therapy is cladribine, administered in two cycles, at 1-year interval. Cladribine inhibits specific signaling cascades, depleting T and B lymphocyte and thus removing autoreactive B and T cells [43]. Cladribine shows a 58% relapse rate reduction and significantly reduces brain atrophy in comparison with placebo [44].

Anti-CD20 therapies like rituximab, ocrelizumab, and ofatumumab deplete B lymphocytes [45], which are effective antigen-presenting cells, and also deplete a small population of T cells. Ocrelizumab was found to be effective in patients with PPMS in a randomized phase III placebo-controlled trial- ORATORIO, in which the percentage of patients with disability progression confirmed at 12 weeks in a time-to-event analysis was reduced by 16% [46]. This can be interpreted as conferring neuroprotection. In the OPERA trials, Ocrelizumab reduced the annualized relapse rate over 2 years by 47% in relapsing MS, compared to IFNβ [47].

Lymphocyte trafficking blockers, whether by modulating the sphingosine 1-phosphate (S1P) receptors in lymph nodes or by blocking the adhesion of leukocytes to endothelial cells by blocking the interaction of the α4-integrin subunit of α4β1 with its counterparts such as VCAM-1 are also high efficacy treatments in relapsing MS. Natalizumab, a monoclonal antibody acting as a α4β1 receptor blocker reduces the rate of relapses at 1 year by 68% as shown in the AFFIRM trial [48]. By preventing CNS inflammation, natalizumab reduces loss of gray matter, and thalamic atrophy [49], prevents microstructural brain damage and has effects indicating an improved white matter microstructure after one-year of treatment [50].

Fingolimod, ozanimod, ponesimod, and siponimod are small molecules that sequester lymphocytes in the lymph nodes [51]. Fingolimod showed some positive effects of remyelination and neuroprotection in several preclinical studies, as it crosses the blood-brain barrier (BBB) due to its lipophilic nature [52,53]. Siponimod also showed a beneficial effect in secondary progressive MS in EXPAND trial in which the proportion of patients with 3-month confirmed disability progression was reduced by 21% [54].

A more recent class of DMTs are Bruton-tyrosine kinase inhibitors (BTK-I), with promising results in preclinical models of MS. Tyrosine kinases are intracellular enzymes that mediate phosphorylation of tyrosine residues contributing to signaling pathways. Bruton tyrosine kinase (BTK) is a cytoplasmic molecule with a signaling role and is expressed by B cells and microglia. These kinases play an important role in the proliferation differentiation, maturation, survival, migration, and activation of the cells in which they are expressed, with an important role in the survival of B cells, microglia, and possibly astrocytes [55]. BTK-I penetrate the CNS. They simultaneously target the adaptive and the innate immune system in the periphery and in the CNS, and therefore are thought to reduce both inflammation and disease progression [55]. Currently, five compounds acting by inhibition of BTK are being evaluated. Results from the evobrutinib EVOLUTION (EvolutionRMS1 and EvolutionRMS2) trials did not meet the primary endpoints and failed to show superiority to oral teriflunomide [56]. Results from the tolebrutinib studies were recently presented at the ECTRIMS 2024 meeting, showing no superior results in relapsing MS when compared to teriflunomide in GEMINI 1 and 2 trials [57] in preventing relapses, but demonstrating a delay in the secondary endpoint of onset of confirmed disability worsening [57]. On the other hand, in the phase III HERCULES trial tolebrutinib delayed the time to onset of 6-month confirmed disability progression by 31% when compared to placebo in non-relapsing secondary progressive MS [58].

The CD40 receptor and its ligand CD40L are part of the tumor necrosis factor receptor superfamily and have important roles in the initiation and maintenance of the inflammatory response [59]. In MS, CD40 and CD40L are expressed both on the immune cells that have infiltrated the CNS and on CNS resident cells, and their interaction maintains inflammation and promotes demyelination [60]. Microglia activated via CD40 by the infiltrating CD40L T cells release cytokines, nitric oxide, and matrix metalloproteinases and this worsens demyelination [60]. Frexalimab is a second generation anti CD40L humanized IgG1 monoclonal antibody which showed positive effects on gadolinium- enhancing T1 weighted lesions at 3 months compared to placebo in a phase 2 trial [61]. The phase III FREXALT trial (NCT06141473) [62] is currently testing frexalimab vs teriflunomide in relapsing MS, while in the phase III FREVIVA trial (NCT06141486) [63] frexalimab is compared with placebo in non-relapsing secondary progressive MS.

Ublituximab is an anti-B cell monoclonal antibody tested in two double-blind, double dummy trials (ULTIMATE I and II) vs teriflunomide in relapsing MS [64]. The trials were positive for Ublituximab with a lower relapse rate and fewer gadolinium-enhancing lesions in the Ublituximab group at 96 weeks [64].

**Figure 2.**
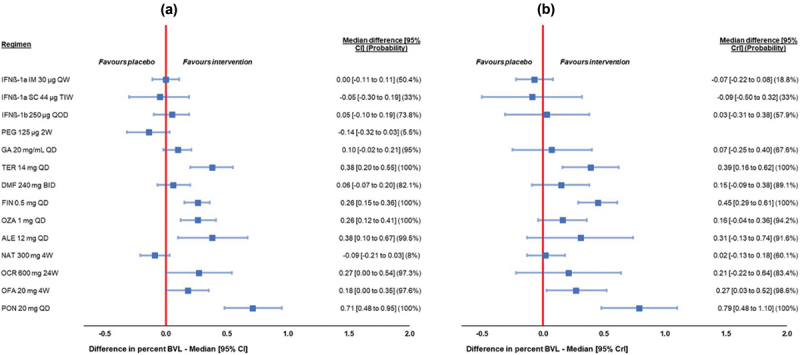
Model-based meta-analysis (MBMA) with adjustment for measurement timepoint and DMT dosage (a), and network meta-analysis (NMA) (b) results for differences in brain volume loss at two years, versus placebo (figure and legend reproduced from Zivadinov et al. [68] with permission from springer nature).

Approved DMTs have effects on brain volume loss (BVL) [65] which can be a measure of their neuroprotective effects. BVL measured with MRI is considered a marker of neurodegeneration, which has been reliably correlated with accumulating physical and cognitive disability [66]. This can be positively influenced by DMTs through various mechanisms which are still investigated [67]. Zivadinov et al. [68] conducted indirect treatment comparisons to estimate the relative efficacy of different DMTs on BVL by the means of both a model-based meta-analysis (MBMA) with adjustment for measurement timepoint and DMT dosage, and a network meta-analysis (NMA). Figure 2 [68] shows MBMA and NMA results for differences in BVL versus placebo at 2 years [68]. The two analysis approaches led to generally comparable results, showing, after adjustment for measurement timepoint and DMT dosage, that sphingosine receptor modulators fingolimod, ponesimod, and ozanimod, and also teriflunomide and alemtuzumab significantly surpassed placebo using BVL as outcome, whereas interferons and natalizumab were not as successful [68].

MBMA and NMA used fixed effect models. Measurement timepoint and dosage were covariates in the MBMA. Round parentheses indicate the probability of being better than placebo. Peginterferon could not be incorporated in the NMA due to a lack of eligible BVL data reported at 2 years.

Abbreviations: 24W = every 24 weeks, 2W = every two weeks, 4W = every four weeks, ALE = alemtuzumab, BID = twice daily, BVL = brain volume loss, CI = confidence interval, DMF = dimethyl fumarate, FIN = fingolimod, GA = glatiramer acetate, IFNβ = interferon β, NAT = natalizumab, OFA = ofatumumab, OZA = ozanimod, PEG = peginterferon β-1a, PON = ponesimod, Q2W = every two weeks, QD = once daily, QOD = every other day, QW = once weekly, TER = teriflunomide.

### 2.2. Cell-based therapies

Cell-based therapeutic strategies are considered to be means for promoting neuroprotection, neurorepair and remyelination, and immune modulation in MS [69]. Stem cells are pluripotent precursor cells and are thought to be able to promote the repair of the neural tissue damage and to modulate immune responses to enhance endogenous repair by several mechanisms, such as secreting neurotrophic factors or by their immunomodulatory effects [70]. Currently, there are no standardized regimens of administration based on their origin (hematopoietic stem cells, mesenchymal stem cells, neural stem cells), their route of administration (mostly intravenous but also intrathecal or intraventricular) or the number of stem cells being administered [71].

Hematopoietic stem cells (HSCT) reside essentially in the bone marrow, are able to develop into any blood cell type, and are indispensable for the homeostasis of the hematopoietic system [72]. In clinical practice, their source for treatment can be either from a donor of cells (allotransplantation) or patients’ own cells (autologous transplantation), with the latter being preferred in auto-immune disease. Stem cell transplantation with autologous hematopoietic stem cells (AHSCT) is one of the strategies proposed to treat active MS. The rationale behind AHSCT is that by using an immune-ablative conditioning regimen, the autoreactive cell clones are depleted with supposedly persistent sustained immune tolerance afterward [69]. The main effect of AHSCT is suppressing inflammation, but possibly stem cells have the potential to migrate into CNS lesions and to induce tissue repair through their postulated capacity to differentiate at low rate into neurons or myelin producing OPCs [73]. Patients that are considered candidates for AHSCT are usually young with an aggressive form of relapsing MS, which previously did not respond to available DMT [74]. AHSCT induces durable improvement in disability in patients with RRMS and prevents disability worsening in the majority of patients [74]. The choice of the conditioning protocol may influence the degree of suppression of clinical and MRI inflammatory activity [74]. The use of AHSCT in patients with active secondary progressive MS is associated with a slowing of disability progression compared with standard immunotherapy, and with a better disability improvement [75]. The ongoing and completed AHSCT trials were recently reviewed [76].

Mesenchymal stem cells (MSC) are non-hematopoietic adult stem cells probably present in almost all of the tissues and are precursors of several types of cells, possibly including neural cells. Based on their source, they can also be classified into several types, with the bone marrow derived MSC being among the most studied ones. They can have immunomodulatory, anti-inflammatory, and paracrine activity, promoting differentiation of neural stem cells and repair in damaged areas. Several studies demonstrate that MSC improve the outcome of experimental autoimmune encephalomyelitis (EAE), the animal model of MS [77]. MSC-derived neural progenitor cells upregulate genes involved in myelination and neuroprotection and downregulate genes involved in inflammation and astrogliosis [78]. Limitations of MSC treatment in MS are the inconstant access of MSC to the multifocal MS lesions and difficulties in monitoring the fate of MSC once they are administered, the adverse effects of CNS inflammatory environment in MS, the lack of standardized methodological approaches and the heterogeneity of MSCs culture, reagents and techniques which raise the need of standard protocols for laboratory management of MSCs to mitigate inconsistency [71]. An interesting avenue is the combination therapy of MSC with neuroprotective, promyelinating, immunomodulatory, and immune reeducating factors, through *in vitro* manipulation of MSCs and cell preconditioning methods [71].

A recent meta-analysis of randomized clinical trials of the efficacy and safety of stem cell transplantation for MS, including both HSTC and MSC, showed that stem cell transplantation can improve the disability of MS patients and reduce brain volume loss and it is generally safe and well tolerated [79].

Neural stem cells are naturally concentrated in specific neurogenic niches and have pluripotent properties, being able to differentiate into neurons, astrocytes, and oligodendrocytes [80]. Their use in clinical trials today is nevertheless difficult due to their limited endogenous reservoir [81], to the laborious isolating and harvesting process and to ethical issues. A recent analysis of 1 year of data from the first group of 15 patients enrolled in an open-label, first-in-human, dose-escalation phase I study of transplantation of allogeneic human neural stem/progenitor cells in secondary progressive MS showed the approach is feasible in humans, and the neural stem cells grafts are tolerated by the MS patients [82]. The number of the injected neural stem cells inversely correlated with parenchymal brain volume changes [82], and longitudinal metabolomics/lipidomics of biological fluids showed time- and dose-dependent responses with increased CSF levels of acyl-carnitines and fatty acids [82].

### 2.3. The blood-brain barrier (BBB)

The BBB is a complex structure of cerebral endothelial cells, pericytes and their basal laminae, astrocytes, and perivascular macrophages [83]. BBB is affected in MS [83]. The disruption of the BBB in MS is proved by MRI techniques and is probably the key event that precedes the intracerebral neuroinflammatory processes [84]. BBB functioning is strictly regulated, and preserving and promoting its integrity can be a neuroprotective strategy. Several DMT have an effect on the BBB. Nevertheless, targeting the BBB is probably beneficial in the early stages of the disease, before CNS intrinsic neuroinflammation and subsequently neurodegeneration start.

The BBB endothelial cells control the migration of leukocytes by chemokines and adhesion molecules [85]. Natalizumab is an example of an approved treatment for RRMS that targets α4β1 integrin and thus blocks leukocyte adhesion to its ligand VCAM 1 (vascular cell adhesion molecule 1) on the BBB endothelial cells [85]. Several other leukocyte cell adhesion molecules play a role in MS and EAE, including CD166 (or ALCAM). These can represent future targets in neuroinflammation and neuroprotection in MS [86].

Leukocytes can have other pathways to pass the BBB and can express alternative adhesion molecules. The Wnt–β-catenin pathway is activated in human MS lesions and in EAE [87]. This pathway promotes the formation of tight junction proteins and transporters in brain capillaries [88]. Dysregulation of the WNT–β-catenin pathway is seen in several neurological disorders, but in EAE, it is responsible for a more severe course of the disease due to an increased trafficking of immune cells into the brain [87]. Modulating this pathway might be a promising therapeutic target for MS neuroprotection.

The integrity of the BBB is maintained due to adhesion and tight junction molecules [89]. Downregulation of claudin 5, a tight junction strand protein, is associated with BBB breakdown in EAE. During a relapse, peripheral blood leukocytes of MS patients have an increased level of claudin 5 [90]. The platelet/endothelial cell adhesion molecule (PECAM1) stabilizes the BBB in vitro, providing neuroprotection in the animal model of MS [91].

Matrix metalloproteinases (MMP) are essential for the leukocyte passage through the BBB in the CNS [92]. MMPs have proteolytic functions whereby they disrupt tight junctions and basement membrane proteins on the BBB, mediating the recruitment of inflammatory cells into the CNS. This role was especially attributed to MMP9 [93]. Pharmacological inhibitors of MMP showed some efficacy in animal models of MS. In relapsing MS, Doxycycline combined with intramuscular IFNβ-1a was associated with low serum levels of MMP9 and with a reduction in brain lesion activity in an open-label study in MS [94].

Endothelial cells produce chemokines, which subsequently promote T cell adhesion in EAE [95]. Chemokine and chemokine receptors are expressed by neurons and other CNS resident cells [96]. Several chemokines and their receptors play a role in the pathophysiology of MS and are potential therapeutic targets. These include CC-chemokine ligand 19 (CCL19) and CXC-chemokine ligand 12 (CXCL12) [95]. Methylprednisolone, a glucocorticoid drug used in the treatment of MS relapses, induces monocyte polarization toward an anti-inflammatory phenotype [97]. Other molecules that show a promising role in promoting neuroprotection through the chemokine pathways are glatiramer acetate and the type 1 interferons [98]. Several antibodies (anti CXCL10 antibody, Mab5261) were tested and showed promising results in the treatment of MS and EAE [99]. By reducing the disruption of the BBB integrity in MS and EAE, these interventions may reduce the cascade of events triggered by BBB dysfunction [100] and hence can have neuroprotective effects.

Another enzyme that influences BBB permeability is angiotensin converting enzyme (ACE) [101]. ACE mediates inflammation and BBB permeability and is increased in MS. Captopril, an ACE inhibitor, improves EAE [102]. ACE inhibitors have been shown to have neuroprotective effects [103].

BBB breakdown is seen less in progressive forms of MS than in relapsing MS, however the dysfunctional neurovascular coupling in progressive MS correlates with gray matter atrophy and cognitive decline, and this suggests that BBB could possibly be a target not only in relapsing MS, but also in the therapeutic management of progressive forms [100].

## 3. CNS inflammation and mechanisms of neuronal injury

BBB breakdown is an early event in MS, even prior to lesion formation, and is associated with pathogenic immune cell infiltration [100]. Acute inflammation in the CNS is supposed to be followed by restoration of local homeostasis, and indeed the immune cells that infiltrate the CNS produce IL10 and IL27, protective cytokines that diminish local neuroinflammation [104]. However, in MS a continuous, chronic, non-resolving inflammation occurs once the BBB is disrupted. T cells, B cells, and myeloid cells that have migrated into the CNS secrete inflammatory cytokines like TNFα, IFNγ, IL4, IL12, IL17 [105]. The activated resident glia will contribute to acidosis, hypoxia, ion imbalance, glutamate excess, and, once the compensatory mechanisms fail, neuronal death. Astrocytes, which normally support neurons, also seem to induce neurotoxicity [106]. The peripheral inflammation is followed by CNS inflammation [14], triggering excitotoxicity and oxidative stress, leading to mitochondrial dysfunction [107] and ultimately being linked to epigenetic consequences [11]. The newly created inflammatory microenvironment has a high energy consumption. The injured neurons release toxic molecules perpetuating the homeostatic imbalance. Continuous tissue inflammation promotes and maintains activation of the surrounding microglia and is responsible for continuous, extended neuronal damage [108].

**Figure 3.**
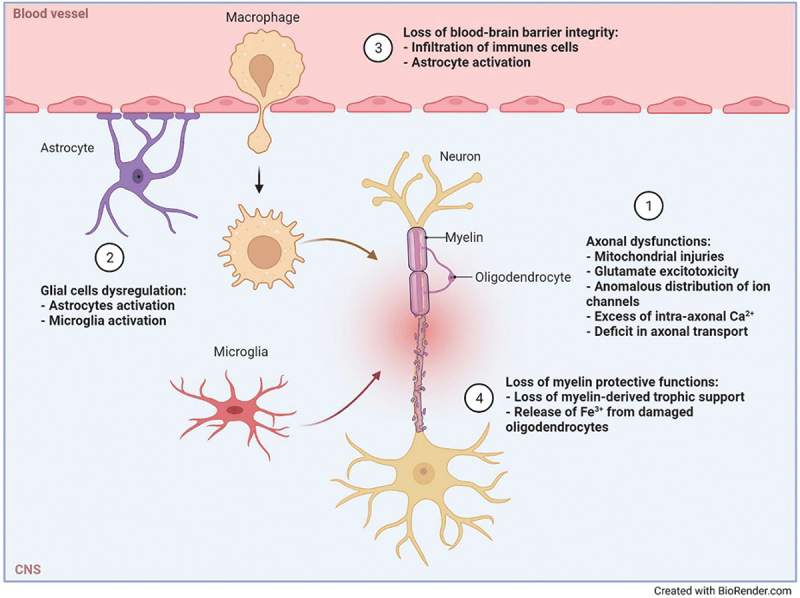
Main targets to achieve neuroprotection in multiple sclerosis (figure and legend reproduced from [25] with permission of Springer nature.

The treatment strategies described above have a role in preventing inflammation and therefore potentially providing neuroprotection [30]. However, almost all approved molecules and those currently in trials fail to play a role in neuron-intrinsic imbalance that finally drives MS pathology and disability [108]. Currently, the concept of smoldering MS has gained a central role in MS, and interest in understanding the principles of neurodegeneration is a priority. Below, we outline the mechanisms underpinning inflammation-induced neurodegeneration and the neuron-intrinsic response to injury, which could be considered targets for neuroprotection strategies (Figure 3) [25].

Drugs under development to achieve a neuroprotective effect have different mechanisms of actions including the promotion of (1) axonal functioning, (2) glial regulation, (3) the integrity of the blood–brain–barrier myelin integrity, and (4) recovery of myelin-protective functions.

### 3.1. Excitotoxicity

The neuronal cell membrane is a barrier between intra- and extracellular ionic environments. In MS, the imbalance in ionic conduction, with the subsequent release of ions and neurotransmitters, leads to axonal degeneration and synapse loss [109]. In EAE, inflammation induces local acidosis and activation of calcium and sodium channels; the excessive accumulation of these ions leads to axonal degeneration [110]. Demyelination leads to persistent activation of voltage-gated sodium channels Nav1.2 and Nav 1.6 [111]. Subsequently, this results in excess intracellular calcium ions, promoting inflammation and contributing to axonal degeneration following demyelination [111,112].

Calcium is one of the most important second messengers in cell signaling. It also controls the neurotransmitter release process in the brain [113]. Calcium concentration is important for oligodendrocyte function, myelin elongation, and sheath development [114]. CNS calcium channels are expressed by different cell types, intracellularly or within the plasma membrane, offering a wide spectrum of potential targets for neuroprotective strategies [115]. Olesoxime, a molecule that binds outer mitochondrial membrane proteins, interferes with calcium homeostasis [116] and promotes remyelination in rodent models [117]. Quetiapine also has a role in calcium homeostasis and has a positive effect on oligodendrocyte development [118].

Nimodipine, an antagonist of the L type VGCC that crosses the BBB and enters the CNS, shows neuroprotective properties through inducing remyelination in animal models [119].

Ion channel modulators have been explored for their potential role in neuroprotection. Sodium channel modulators, phenytoin, carbamazepine, lamotrigine, and amiloride, have been tested with some positive effects in animal models and some small clinical trials [120]. Potassium channels have been implicated in the progression of MS. In the cuprizone model of demyelination, immunohistochemical staining of mouse brains shows high expression of the potassium channel isoform Kv1.3, and modulating Kv1.1 and Kv1.3 correlates with myelination [121]. Potassium channel regulators (aminopyridine, diazoxide) have also been considered neuroprotective; however, the results with their use have had conflicting, besides their symptomatic effect [122]. In an astroglial cellular model of inflammation, 4-aminopyridine exacerbated inflammation through release of pro-inflammatory cytokines [121].

In parallel with ion imbalance, there are other pathways contributing to excitotoxicity. Glutamate is released by dying neurons and is elevated in the CSF and brains of people with MS [123]. This leads to overactivation of ionotropic glutamate receptors (GRI) N-methyl-D-aspartate receptors (NMDAR) and AMPAr [124]. The overactivation of glutamate receptors increases the intracellular calcium, amplifies excitotoxicity, leads to mitochondrial dysfunction [125] and promotes cellular death [124]. Increased extrasynaptic NMDA receptor signaling contributes to a deficient nuclear calcium signaling [126]. Moreover, another NMDAR-dependent activation induces uncontrolled calcium release from calcium storage and subsequently generates calcium-dependent cell death [127]. Considering this, AMPAr and NMDAR antagonists might be seen as potential therapeutic targets in promoting neuroprotection. The AMPAr and NMDAR antagonists improved neuronal loss in experimental autoimmune optic neuritis [128]. The importance and complexity of these mechanisms is exemplified by a selective inhibition of the AMPAR subunit Glu4 in oligodendrocytes, which improved the course of EAE [129]. Both receptors are part of the GRI signaling. However, the long-term use of these antagonists might be deleterious, as GRI are also expressed in oligodendrocyte progenitor cells (OPC) and oligodendrocytes, and their blockade can interfere with remyelination [130,131].

Riluzole acts by reducing the glutamate release, inhibits NMDA receptors and reduces axonal damage in EAE [132]. In a pilot study conducted on people with MS, riluzole showed a reduction in the rate of cervical cord atrophy and the number of new T1 hypointense lesions [133]. The MS-SMART phase 2 a multicentre, multi-arm, parallel group, double-blind, randomized placebo-controlled trial assessed the neuroprotective effects of riluzole, amiloride, and fluoxetine. When compared to placebo in people with SPMS, none of these drugs had a significant effect in reducing progression [134].

Ifenprodil is an allosteric negative modulator interacting selectively with the GluN2bR subunit of the NMDA receptor, blocking the agonistic excitatory effect of glutamate [135]. Infenprodil is currently tested as a remyelinating drug in relapsing MS (NCT06330077).

### 3.2. Mitochondria dysfunction and energy failure

Mitochondria are an important source of oxygen and nitrous oxide production and have an important role in maintaining intracellular calcium homeostasis [136]. The neuronal mitochondrial respiratory chain is impaired and shows reduced oxidative phosphorylation in EAE and in people with MS [137]. This affects the capacity of neurons to generate ATP. Energy failure results in parallel with an increased energy demand in the demyelinated axons [138]. Mitochondrial dysfunction precedes clinical onset and axonal loss in EAE, potentially making its role essential to neurodegeneration [139]. Persistent oxidative stress and continuous inflammation exposure of the neuron contribute to the impaired mitochondrial activity [140]. Several intrinsic molecular alterations in neuronal cell bodies reflect mitochondrial dysfunction [141]. Kinesins, proteins essential to the transport of the mitochondria from the neuronal soma to the axon are also impaired in MS and contribute to axonal energy failure [141]. As MS evolves, mitochondrial dysfunction is a process that is continuously worsening, being promoted by demyelination, iron accumulation, and oxidative impairment [142].

The pro-oxidative environment in progressive MS is compounded by deficiency of glutathione, which is one of the most important antioxidants in the brain [143]. Due to its central role as antioxidant, glutathione has been proposed as an interesting tool in preventing neurodegeneration [144]. In progressive MS, there is significantly lower glutathione compared to RRMS, and this suggests a more important role of oxidative stress in the progressive stages of MS [145]. Glutathione is also a possible biomarker of cognitive impairment in MS [146].

Compounds that support cellular metabolism (vitamin D, iron, high dose biotin) have been suggested as possible treatments in progressive forms of MS [147]. Biotin is a cofactor for five carboxylases involved in fatty acid synthesis and energy production. High doses of biotin reduced the progression of disability in a phase 2 trial but failed to show efficacy in a phase 3 trial [148].

### 3.3. Epigenetic dysregulation

Neuroinflammation can affect nuclear transcription via epigenetic alterations. Epigenetic regulation occurs through altering availability of DNA for initiation of transcription by changes such as phosphorylation, methylation, or acetylation of DNA or histone proteins [108]. Neuronal epigenetic modulators are dysregulated in animal models of MS and in humans with MS [149,150]. DNA methylation is modified in people with MS, independently of their genotype [9]. B cells and monocytes have a differential methylation from disease onset [151]. Monocytes might be a future therapeutic target in MS [9], as B cells are today. The methylome profiles of cell-free neuronal DNA in the blood or CSF may represent a new prognostic measure of neurodegeneration [152]. miRNAs can act as epigenetic controllers and possibly be relevant to neuroprotection. For example, miR-92a is part of a miRNA–mRNA axis with neuroprotective role in neuroinflammation-induced neurodegeneration [153] in EAE. More study is needed to understand how epigenetic players can be used to monitor neurodegeneration or to achieve neuroprotection.

### 3.4. Neuronal immune signalling and the microglia – astrocyte crosstalk

The CNS inflammatory environment in the MS brains creates stressors that lead to changes in neuronal metabolism and excitability. In turn, neuronal immune signaling, which is part of the neuron’s stress response, participates in modulating the inflammatory milieu [108]. Neuron-specific profiling of EAE mice and studies on brains of people with MS show strong intrinsic neuronal immune signatures like those of neurotropic infections [154,155]. CNS infiltration by neutrophils and lymphocytes, especially CD8+, Th1/17 and Th2 subsequently activates microglia and contributes to local immune response and signaling by secreting IFNγ, IL17, and IL4 [156]. The neuronal inflammatory response modulates the CNS infiltration and activation of leucocytes by secreting reelin [157], matrilin2 [158], CCL2 [154] and Salm5 [159]. Reelin, a glycoprotein that guides neurons during brain development and is a synaptic homeostatic regulator, regulates the NF-κB mediated expression of vascular adhesion molecules [160]. Reelin depletion reduces vascular adhesion markers, reducing endothelial permeability and therefore CNS leukocyte infiltration [157]. On the contrary, matrillin 2 exacerbates neuroinflammation, inducing proinflammatory response in EAE models [158].

Microglia and monocyte-derived macrophages have an important role in CNS clearance of degraded material in physiological conditions [161], but when overactivated can worsen neuroinflammation and promote neurodegeneration [162]. There are two described microglial profiles – one proinflammatory (M1) and another pro-repair (M2) [163], which suggests that M2 microglia have an endogenous repair potential [140]. Astrocytes are abundant glial cells and have a central role in neuroinflammation. In vitro models suggest a possible neuroprotective role of astrocytes in MS, through suppressing inflammation and increasing the expression of neurotrophic factors [164]. Through different cytokines and chemokines, there is crosstalk between astrocytes and microglia, which either limits or promotes neurodegeneration, and understanding the pathways of this crosstalk could eventually help in preventing neurodegeneration and promoting neuroprotection [165]. Molecules produced by microglia and effector T cells promote astrocyte activation which in turn increases microglial activation, recruitment of pro-inflammatory monocytes, and the production of neurotoxic factors, while regulatory T cells and possibly NK cell-derived cytokines promote astrocyte anti-inflammatory activity [165]. A comprehensive understanding of neuronal immune signaling, its interaction with the microglial environment and its consequences on CNS homeostasis could provide new therapeutic approaches and targets to be used in promoting neuroprotection.

Ibudilast is a nonselective phosphodiesterase inhibitor which has anti-inflammatory and neuroprotective effects in animal models of MS [166] and was tested in phase 2 trials in MS [167]. Ibudilast does not effectively decrease focal inflammation in relapsing MS, but has an effect on preserving brain volume and decreases the volume and improves magnetization transfer measures of slowly expanding lesions [167]. Ibudilast may have a role in the treatment of progressive MS. Ibudilast may reduce neuronal degeneration through inhibition of astrocytic and microglial TNF-alpha release and of astrocyte apoptosis [167].

### 3.5. Promoters and amplifiers of neurodegeneration

While the initial disruptive signals of homeostasis are external to the CNS, neurodegeneration is amplified by internal signals present in the impaired neurons [108]. These involve dysregulation of axonal transport, protein aggregation, and the unfolded protein response in ER.

#### 3.5.1. Axonal transport dysregulation

Energy supply in neurons is dependent on transport of organelles and structural proteins [168]. As previously discussed, energy supply failure and mitochondrial dysregulation have a central role in neurodegeneration; they directly impact neuronal transport [169], which in turn promotes further neuronal damage. Studies in EAE show a widespread transport deficit in the axons during acute inflammation [170]. Axonal transport deficit precedes structural impairment of axons, cargos, and microtubules [170,171]. Possibly at this point axonal injury might be reversible and anti-inflammatory interventions could prevent the distal organelle supply failure seen in advanced stages of MS [171]. Dysregulated axonal transport has been described in relation to several neuronal transport proteins. KIF5A is an axonal motor protein responsible for anterograde transport. Its expression is reduced in normal appearing white matter [172] and has an important role in ongoing neurodegeneration in MS. The stability of microtubules (MT) is also important for neuronal transport mechanisms. The transmembrane protein dysferlin, implicated in senescence pathways, which are accelerated in progressive MS and relate to disease severity [173], is linked to regulating MT stability, and may be involved in modulating membrane repair and neuronal survival [108].

In theory, restoring axonal transport could promote neuroprotection, but more study is needed to understand the key processes, which can allow designing a meaningful intervention.

#### 3.5.2. Neuroaxonal injury and protein aggregation

Protein aggregation plays an important role in MS pathogenesis. Proteasomal inhibition allows accumulation of misfolded proteins. During neuroinflammation in MS the proteasome activity is compromised [174]. In mouse models of MS, and in patients with MS [149], inflammation leads to neuronal somatic upregulation of the presynaptic structural protein bassoon (BSN). BSN accumulation has a very strong neurotoxic potential; pharmacological proteasome activation triggers BSN clearance and promotes neuron survival [149]. BSN accumulation leads to tau-seed propagation and neurotoxicity, underlying its role in neurodegenerative diseases [175]. It is not clear whether the entire BSN protein, or only several of its fragments, aggregate in the soma. Removal of abnormal protein deposition could represent a therapeutic target in preventing neurodegeneration, however its dynamics is not yet completely understood. PROTAC (proteolysis-targeting chimera) is a molecule that acts by mediated targeted protein degradation [176]. Its use in neurology is limited by its low CNS penetration and by the fact that BSN is not accessible in its presynaptic state.

The protein degradation pathway is upregulated in neurons during inflammation. The UPS (ubiquitin – proteasome system) protects the cells against protein cell load, and leads to proteasome proteolytic degradation [177]. Targeted protein degradation is an emerging therapeutic strategy that has been used in other diseases such as cancer [178].

#### 3.5.3. Unfolded protein response in ER

The endoplasmic reticulum (ER) is interconnected with the nuclear membrane. ER has an important role in cell homeostasis by protein secretion and folding, lipid biosynthesis and calcium homeostasis [179]. The unfolded protein response (UPR) is the ER response to the stress caused by inflammation and by accumulation of unfolded or misfolded protein aggregates. UPR initial adaptive role is aimed at restoring homeostasis but when chronically overactivated it induces apoptosis and therefore accelerates neurodegeneration [179]. In experimental animal models of MS (EAE and cuprizone), there is an important role for UPR activation in oligodendrocytes [180]. Protein kinase R-like endoplasmic reticulum kinase (PERK) detects and responds to imbalances in protein folding in the ER [181]. Moderate activation of the PERK branch of UPR protects oligodendrocytes from inflammatory attacks, proving the importance of ER in neuroprotection [182].

## 4. Neuronal cell death

The mechanisms discussed above finally determine the neuronal cell fate. Several types of neuronal cell death have been described in neurodegenerative diseases and in MS. Experimental data suggest that a certain degree of neuronal cell death might even precede the CNS inflammation [183].

### 4.1 Apoptosis

The role of programmed cell death (apoptosis) in neuronal cells (oligodendrocytes and neurons) and its role in MS pathogenesis needs further characterization as there is little proof of it in EAE and MS models [184]. Sildenafil improved disease course in EAE models [185], by decreasing apoptosis via the modulation of caspase expression.

### 4.2 Necroptosis

Necroptosis is one of the best-characterized cell death pathways in MS. TNFα, a proinflammatory cytokine, is elevated in the cerebrospinal fluid (CSF) and serum of patients with MS and plays an important role in intrathecal inflammation and axonal neurodegeneration [186]. Its role is particularly important in the gray matter. Necroptotic proteins resulting from TNF pathway in neurons are increased in MS cases with prominent meningeal inflammation [187].

Moreover, TNF and its soluble receptors are associated with disability progression in MS [187]. TNF α induces programmed necrosis and inflammatory death in oligodendrocytes and in neurons, through one of the best-characterized necroptotic pathways [188]. Meningeal B cell follicular structures constantly secrete TNF and in vivo studies show focal cortical thinning next to the leptomeningeal inflammation, promoting neurodegeneration [189]. Transcriptomics analysis of gray matter tissue blocks showed several changes at protein level in TNF signaling pathways [190]. Dysregulation of several proteins, including the key proteins RIPK1, RIPK3 and MLKL is a hallmark of neurodegeneration [191]. Activation of TNF/TNF receptor signaling 1 pathway finally leads to protein characteristics of necrosome [190]. Binding of the soluble TNF to the TNF receptor 1 (TNFR1) controls pro-survival and cell death pathways [192]. Transmembrane TNF (tmTNF) mainly regulates cell survival and regeneration when binding to TNF receptor 2 (TNFR2) [193].

The necroptotic cell death pathway is regulated by RIPK1 (Receptor interacting serine/threonine protein kinase), RIPK3 and MLKL (mixed lineage pseudokinase ligand) under caspase-8 deficient conditions [194]. These are the hallmark of necroptosis [191]. Targeting RIPK1 is a promising pathway in preventing neurodegeneration [195]. In animal models of MS (EAE and cuprizone) TNF mediated pathway induced neurodegeneration via death of oligodendrocytes, and RIPK1 inhibition induced oligodendrocytes survival [191].

Moreover, inhibition of RIPK1 by ZJU-37 promotes oligodendrocytes progenitor proliferation and remyelination via NF-κB pathway [196].

However, blocking the TNF-alpha immunobiological pathway with antibodies may not be an appropriate strategy in MS. TNF-alpha is highly pleiotropic and its actions in animal models of MS and MS are not restricted to promoting inflammation, but its downstream induction of anti-inflammatory factor counters its own pro-inflammatory effects [197]. Anti-TNF-alpha treatments showed mixed results in EAE, whereas in trials in MS it was detrimental [198,199]. In view of the need to at the same time preserve its many helpful functions, the challenge of modulating selectively the TNF pathway for neuroprotection is that to account for the overlapping biological functions of TNF-alpha/TNFR with that of other ligand/receptor superfamily members [200] and the presence of genetic polymorphisms in individuals with MS.

### 4.3 Ferroptosis

Ferroptosis is an iron-dependent cell death pathway and has an important role in MS progression [201]. Ferroptosis contributes to T cell and phagocyte activation and drives oligodendrocyte and neuronal death [202–204]. In MS and EAE, glutathione peroxidase 4, which is the main inhibitor of ferroptosis, appears to be suppressed [150]. Inhibition of ferroptosis is a promising strategy for promoting neuroprotection in MS [205], even if the neuronal ferroptosis pathway is not completely understood. Iron chelation molecules, like deferiprone (DFP) show an anti-inflammatory effect and myelin protection in demyelinated optic nerve [205].

Considering the link between ferroptosis and the oxidative stress, lipoic acid (an endogenously produced antioxidant) and N-acetyl cysteine (which is the precursor of glutathione) are currently tested in MS patients. Alpha-lipoic acid, a natural antioxidant of the human body showed a positive effect on the whole brain atrophy in people with secondary progressive MS, with a good safety profile [206], and has immunomodulatory effects in several other autoimmune diseases [207]. The LAPMS trial (NCT03161028) is a phase two placebo-controlled study testing whether lipoic acid is effective in preserving mobility in people with progressive MS. NACPMS (N-Acetyl Cysteine as a Neuroprotective Agent in Progressive Multiple Sclerosis) trial is a double-blind placebo-controlled trial that evaluates the effect of N-acetyl cysteine (NAC) on MRI progression [208]. A previous study testing NAC showed an increased glucose metabolism in MS patients and correlated with an improvement in cognitive symptoms [209].

Recently, it was shown in a mouse model of MS that the stimulator of interferon genes (STING) connects inflammation with glutamate-driven excitotoxicity to induce ferroptosis [210]. This mechanism of inflammation-induced neurodegeneration possibly can represent a target for treatment [211].

## 5. Remyelination

In addition to damage caused by immune attack and neuron-intrinsic molecular mechanisms, there is incomplete and inefficient remyelination in MS.

It is currently presumed that demyelination is the most prominent feature in MS physiopathology [212].

One strategy to prevent neurodegeneration and promote neuroprotection is to enhance myelin regeneration, restore nerve conduction, and ensure the axonal metabolic support [213]. Myelin is responsible for axonal saltatory transmission. It also provides metabolic support to axons by supplying phosphatidylcholine, N-acetyl aspartate (NAA) [214]. Preventing demyelination and promoting remyelination can restore function in several animal models [215]. Evaluation of remyelination is nevertheless challenging in human clinical trials [216].

Oligodendrocytes, the myelin-producing cells of the CNS, provide support to axons by producing lactate, a mitochondria fuel, and by exchanging potassium ions [217] and contributing to potassium clearance.

The cells responsible for remyelination are the oligodendrocytes or the OPC, their progenitors which contribute to myelin formation during the embryonic stage [213]. The process of remyelination is complex and requires activation, migration, and differentiation of the OPCs into mature oligodendrocyte [218].

Initial strategies in enhancing endogenous remyelination were explored by promoting migration, proliferation, and differentiation of OPCs [219]. Recently, it has been observed that mature oligodendrocytes surviving within a demyelinated area are also able to generate new myelin sheaths [220].

One of the most commonly proposed strategies in remyelination is promoting the differentiation of the OPCs into mature myelin-producing cells [220]. Many groups have focused on finding pathways and screened small molecules that could promote OPC differentiation [221,222].

Clobetasol and miconazole have an effect on glucocorticoid receptor signaling and mitogen-activated protein kinase (MAPK), respectively, and promote remyelination in vivo through these actions [223].

Myelin itself inhibits OPC differentiation [224]. Myelin-mediated inhibition of OPC differentiation is controlled by either RhoA-ROCK-II (Rho kinase) pathway signaling or protein kinase C (PKC) pathway signaling. Inhibition of either of these two pathways promotes OPCs differentiation in the presence of myelin [224].

Epigenetic processes such as DNA methylation appear to regulate the factors involved in OPC differentiation [225] and this could provide new possibilities for promoting neuroprotection.

Metformin is an agent that decreases DNA damage and promotes metabolic functions. It showed regenerative properties in rodents and is tested currently in a clinical study – MACSiMiSE-BRAIN, a multi-center two-arm, placebo-controlled clinical trial in people with non-active PMS [226].

Another explored target in remyelination is Opalin (TMEM 10), a mammalian-specific CNS myelin gene that is expressed by oligodendrocytes in human remyelinating MS plaques [227].

Anti LINGO1, a monoclonal antibody that acts by blocking the leucine-rich repeat immunoglobulin-like domain containing protein 1 (LINGO) also seems to promote remyelination [228].

Lipid and cholesterol synthesis is essential for myelin formation, so cholesterol supplementation has also been explored as a therapeutic intervention [229].

Modulating estrogen receptors with Tamoxifen when administered to mice demonstrated an increase in remyelination [230].

Clemastine fumarate, an oral antihistamine drug and muscarinic receptor antagonist, reduced the latency of visual-evoked potentials in chronic optic neuritis [231] therefore showing some promising remyelinating effects. The efficacy of clemastine fumarate was confirmed in the 2017 Re-BUILD trial, which met the primary end point indicating remyelination in vivo [232]. One of the suggested pro-myelinating mechanisms of clemastine and metformin is modulating the OPC states through modulating NMDAR surface expression on OPC [233]. OPCs have different proliferation and differentiation features depending on brain region and age and these properties correlate with the cellular density of voltage-gated ion channels and glutamate receptors [233]. Clemastine and metformin extend the window of NMDAR surface expression, promoting an NMDAR-rich oligodendrocyte precursor cell state, and this can be a possible pro-remyelinating mechanism [233].

The depletion of OPCs in chronic long-standing MS patients provides a probable explanation for the limited remyelination in these patients [234]. The capacity of OPC for differentiation and proliferation is altered in MS. This has been demonstrated in aged rodents [235].

There is a large spectrum of molecules that target OPC differentiation and migration that fail to prove effective in the final process of remyelination [236]. This is probably due to the limitations of these molecules to act effectively in the complex interplay of local cellular and immune actors [237]. Their remyelinating actions may be neutralized or reversed by coexisting inflammatory processes. In addition, restoring myelin requires the preservation of the axon, so this strategy is ineffective when axons are lost.

## 6. Axonal regeneration

Axonal loss is the principal unsolved cause of irreversible disability in people with MS, and preventing it remains a major challenge. Reducing inhibitory signaling on axonal growth is one of the most explored pathways in promoting axonal survival [238]. Neurite outgrowth inhibitor A (Nogo-A), has been explored its role in CNS plasticity. In animal models, it has been established that by inhibiting it or its axonal receptor LINGO-1 [239], axonal repair is promoted [240]. Nevertheless, after the promising results in animal models, the AFFINITY trial [241] and SYNERGY trial [242] failed to confirm the positive results of Opicinumab (a fully humanized anti-LINGO-1 antibody), showing no significant improvement in disability compared with placebo in patients with relapsing MS.

One explanation of failure in trials of neuroprotective or remyelinating drugs in MS could in part be due to the limited capability of large molecules to reach the CNS. Recent data from animal studies suggest that the nasal administration (via olfactory mucosa) of Nogo-A-neutralizing IgG can improve EAE (motor symptoms and preservation of myelin in the spinal cord) [243], and this mode of administration could possibly be used in MS.

Repulsive guidance molecule A (RGMa) acts in the CNS by binding to the Neogenin receptor [244], inhibiting neurite growth. Several trials with antibodies that block RGMa showed a clinical improvement in rodent models and non-human primates [244,245]. Its effects have been observed in stroke and spinal cord injury models where neurodegeneration mechanisms may be different from those in MS [246]. Elezanumab, a fully human monoclonal antibody directed against RGMa was tested with a good safety profile in a phase 1 clinical trial in MS patients and healthy controls [247]. It increased CSF IL-10, an anti-inflammatory and potentially neuroprotective cytokine.

## 7. Lifestyle factors and neuroprotection in MS

In MS, as well as in other neurodegenerative conditions, the brain reserve is directly influenced by lifestyle factors contributing to the neuronal vulnerability, and subsequently to disease progression. Lifestyle and environmental factors have been identified as key contributors in MS susceptibility and MS progression [8,248]. Whilst exercise and healthy diet can have anti-inflammatory effects, their specific neuroprotective effects (independent of immune modulation) in people with MS are more difficult to demonstrate but are suggested by evidence from animal models.

Endurance exercise has neuroprotective effects in animal models of MS [249]. The mechanisms by which exercise can be neuroprotective in animal models of MS were reviewed extensively [250,251]. These include neurogenesis, remyelination [252] and OPC recruitment [253] reducing oxidative stress leading to neuron survival [254], reduced microglia activation [255], and stimulating neuronal regeneration through inhibiting several transcripts related to the Nogo inhibitory pathway [256], and increasing neurotrophic factors such as brain-derived neurotrophic factor (BDNF) [257]. However, demonstrating neuroprotective effects of exercise in people with MS is less clear. A meta-analysis of studies on effects of exercise training on Neurotrophic Factors in people with MS showed that that exercise improves chronic levels of BDNF, with less convincing evidence for acute levels or other neurotrophic factors [258]. An important observation is that remyelination induced by exercise in a mouse model was augmented when combined with treatment with clemastine [252], with enhancement of axon survival and remyelination. This suggests that further trials in humans, which combine the two interventions -exercise and a neuroprotective/remyelinating agent, could be considered. However, translating these findings to clinical practice in people with MS is complex, due to inter-species differences, the multifactorial nature of MS, and the need for the interventions to be tailored to individual capacities and disease stages.

Mediterranean diet (MD), known for its benefits in cardiovascular diseases and several other chronic conditions, also shows some potential benefit in MS course [259]. MD is associated with less objective disability in MS [260].

Fatty acids are required as raw material for myelination. Their pro-remyelinating effects in animal models were recently reviewed [261]. Omega-3 fatty acid-enriched diet is thought to have some positive effects by stimulating myelin gene expression [262]. Interestingly, the dietary substrate of *N*-3 polyunsaturated fatty acids (PUFAs) may be relevant to remyelination: in the cuprizone animal model, a fish-rich diet may offer a protective role in demyelination whilst no effect of a cod liver oil-based diet was noted [263]. The authors suggest that the source of *N*-3 PUFAs, or other components in the fish, may be relevant, and this may explain discrepant results in dietary intervention studies for demyelinating diseases [263].

The bidirectional communication through the gut-brain-axis is accepted as being part of the brain physiology in health and disease, although the full complexity of this interaction is not yet completely understood [264]. Brain neuroinflammation in MS is influenced both by microbiome-derived metabolites or by microbiota-derived systemic signals [265–273], with a loss of immune tolerance which is age-dependent [274]. How successful the treatment strategies targeting gut microbiota in MS could be (such as dietary interventions, fecal microbiota transplantation, bacterial metabolites supplementation) should be tested in controlled clinical studies which should include fully phenotypically characterized MS patients and would specifically address different time points and stages during MS course [268].

Several dietary interventions with neuroprotective goals were suggested in MS [275]. For example, the MIND (‘Mediterranean-DASH Intervention for Neurodegenerative Delay’) diet which aims to combine the antioxidant and anti-inflammatory features of the Mediterranean Diet and the DASH (‘Dietary Approaches to Stop Hypertension’) is suggested to have clinical and biochemical positive effects in MS patients, through an improvement in the lipid profile, and a decrease in inflammation and oxidative stress [276]. There is a need for standardization in dietary interventions in MS, which can be facilitated only through their investigation in large prospective clinical studies.

The role of antioxidants in neuroprotection and cognition in MS is unclear. A recent systematic review of studies on the association of serum levels of inflammation and oxidative stress markers with cognitive outcomes in MS was inconclusive [277]. However, a recent systematic review investigating the outcomes of CoQ10 supplementation in MS suggested that CoQ10 possibly could exert dose-dependent beneficial effects on ameliorating oxidative stress in MS, and possibly could positively impact symptoms such as fatigue and depression, with the caveat of the limitations of the small number of included studies [278]. (Poly)phenols are a wide and heterogeneous class of substances with several potential health benefits. A questionnaire study on 106 MS patients regarding the possible association between total and individual (poly)phenol intake, major dietary sources, and the severity of MS suggested a higher intake of hydroxycinnamic acids and vegetables may impact MS severity [279].

Several studies have reported lower levels of uric acid, a recognized antioxidant, in people diagnosed with MS compared to healthy people, leading to the hypothesis that higher uric acid levels might reduce the risk of developing MS [280,281]. However, a Mendelian randomization study demonstrated that genetic predisposition to lower uric acid levels had no impact on MS risk [282]. A very recent nationwide case-control study using Danish health registry data explored the association between allopurinol use in the 5 years preceding the diagnosis of MS and the risk of MS, suggesting an inverse relationship between uric acid levels and MS risk [283]. The authors suggested reduced uric acid levels may be linked with early MS development [283], possibly due to its antioxidant properties (they did speculate that uric acid in MS may be depleted, as it scavenges nitrogen and reactive oxygen species [284]), or alternatively possibly due to a potential effect of allopurinol against MS [283].

The results of a systematic review on the role of lipoic acid (LA) supplementation in MS including in vitro and in vivo LA studies (20 studies on cell and animal models of MS, 12 studies in people with MS) [285] suggested that LA may have neuroprotective effects and has generally positive effects in EAE positively impacting the clinical disability scores, and that LA is well tolerated and possibly with a suggestion of clinical benefit on walking performance in people with MS; however, this positive outlook should be mitigated by considering the heterogeneity of studies, dosing, and methodologies [285].

Curcumin, a natural phenolic antioxidant, ameliorates significantly the loss of myelin and reduces astrocyte activation in the corpus callosum in the cuprizone mice model mice and in primary astrocytes stimulated with lipopolysaccharide [286].

Vitamin D has antioxidant abilities, however in people with MS oral vitamin D supplementation are probably insufficient to induce a beneficial effect on the pro- and antioxidant balance [287]. Vitamin D in MS may have pleiotropic immune effects which include promoting regulatory cellular subsets while decreasing differentiation of T or B effector cells, and reducing astrocyte and microglia activation [288]. A very recent randomized clinical trial showed that high-dose vitamin D monotherapy commenced within 90 days after the occurrence of first MS relapse reduced disease activity significantly compared with placebo, with most benefit in patients with severe vitamin D deficiency (<30 nmol/L) [289]. However, the role of high-dose vitamin D therapy in reducing progression in late MS is unclear and not yet fully proven.

The interaction between genetic and environmental factors is crucial to MS pathogenesis. As mentioned above, epigenetic mechanisms play a role in neurodegeneration in MS, and environmental factors may engender epigenetic influences. Epigenetic biological aging in MS can be measured through different ‘epigenetic clocks’ which do reflect specific pathophysiological features of MS [290]; such an epigenetic clock indicates accelerated aging in glial cells of people with progressive MS [291]. Cigarette smoking is associated with the risk of MS [292] and importantly with more severe MS and faster disability progression [293]. Smoking in MS induces epigenetic aging of lung bronchoalveolar lavage cells [294], and smoking induces DNA methylation alterations with exposure-response relationship in people with MS, especially in the group with major genetic risk factors for MS [295]. Smoking cessation is associated with a reduced risk of disability progression, and therefore it should be proposed to patients as a neuroprotective strategy [296,297].

By combining genome-wide association studies (GWAS) with data on chromatin accessibility and histone modification in immune and brain resident cells, Ma et al. showed that MS GWAS-associated loci are enriched in open chromatin regions in B cells and microglia [298]. Regarding the propensity for MS progression, a possible genetic-epigenetic interaction in the 1q21.1 locus could mediate the development of primary progressive MS [299].

The Epstein-Barr virus (human herpesvirus 4), infection is strongly associated with MS risk, playing a critical role in MS pathogenesis and possibly, through reactivation, in MS progression [8,300]. By preventing the deleterious cascade of events EBV triggers and maintains, one could argue that the vaccination against EBV [301] can represent a strategy for neuroprotection in those individuals at risk. Human Endogenous Retroviruses (HERVs) are endogenous viral elements of the human genome whose expression is associated with MS [302] and have the potential to activate inflammatory immunity [302]. HERV biological effects in MS may involve innate immune pathways activation by the envelope protein of MSRV (MSRVEnv) [303] and could promote progression toward MS. Interestingly, persons infected with the human immunodeficiency virus (HIV) may have a lower MS risk than healthy people, possibly due to HERV expression suppression by antiretroviral therapies [304]. The negative association between HERVs and circulating vitamin D [305] on one hand, and the inverse correlation of vitamin D levels with EBV load [306] and the ability of EBV to act as a potential superantigen transactivating HERVs [307], suggests these factors could interact [308].

Identifying and treating infections and comorbidities might also be used as a neuroprotective strategy. People with MS have a higher overall risk of infections that subsequently contributes to symptom worsening [309,310]. Cardiovascular comorbidities also have an impact on disease prognosis and evolution, and it is therefore relevant to prevent and effectively control vascular risk factors in people with MS [311].

## 8. Fluid and radiological biomarkers for progression and neurodegeneration

**Figure 4.**
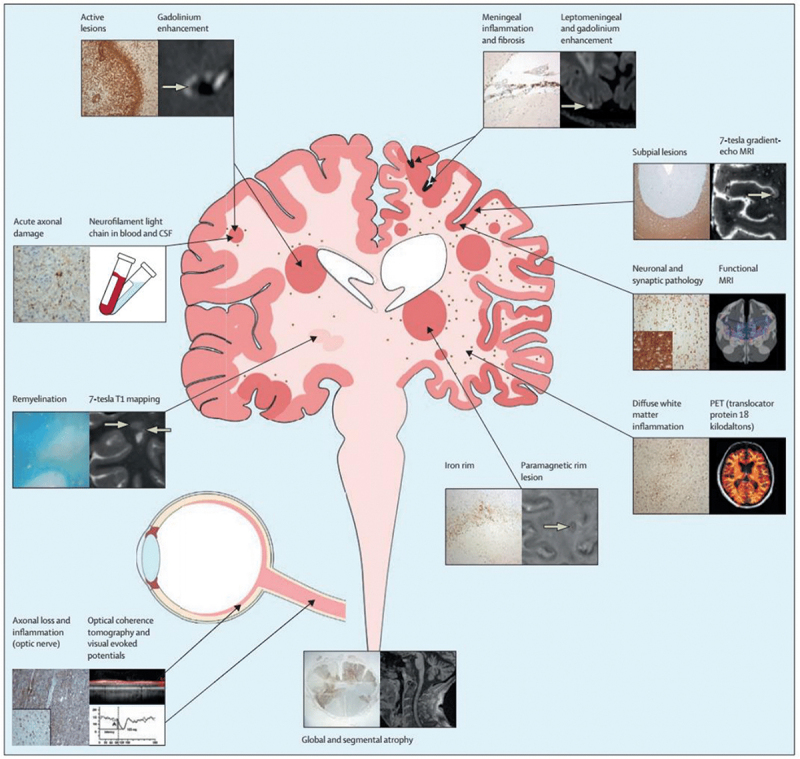
Mechanisms of injury and compensation and associated measures in multiple sclerosis (figure and legend reproduced from Kuhlmann et al. [4] with permission from elsevier).

The accurate measurement and monitoring of neurodegeneration and axonal loss in MS progression is challenging. Both fluid and radiological biomarkers are tools used to capture and monitor the processes of injury and compensation in MS [4], which vary during disease course (Figure 4) [4]. The role of MS fluid biomarkers has expanded significantly beyond the initial diagnostic purpose of oligoclonal bands (OCBs) or of the κ-free light chain index [312]. Neurofilament light chain (NfL) is a marker of axonal injury and a biomarker for disease activity and progression, with both CSF and serum levels correlating with relapse rates, brain atrophy, and long-term disability outcomes [313]. Markers of astrocyte and microglial activation such as glial fibrillary acidic protein (GFAP) and chitinase-3-like protein 1 (CHI3L1), provide insights into compartmentalized inflammation and neurodegeneration [314,315]. However, these biomarkers face significant limitations for clinical translation due to intra-individual variability, age-related changes, and comorbid conditions that can alter biomarker levels and reduce their specificity [312].

Early in the disease (left side of the figure), injury caused by focal lesions and associated axon damage can be compensated for by mechanisms such as remyelination. Over time, lesions in the gray and white matter and axon damage accumulate; meningeal inflammation, diffuse microglial activation in the extralesional white matter, and slow expansion of existing lesions also become more prominent (right side). Progression is further driven by decreased remyelination capacity and damage to neuronal networks mediated by loss of neurons and synapses. Ongoing low-level inflammation and loss of compensatory mechanisms result in segmental and global atrophy. The histological panel depicting the optic nerve shows axon neurofilaments, whereas the inset shows CD68+ myeloid cells. The visual evoked potential trace depicts delayed latency, indicating slow conduction related to demyelination. Neuronal and synaptic pathology can be detected by reduced staining for NeuN, a marker for neurons (panel), and synaptophysin, a marker for synapses. The blue lines in the radiological image symbolize neuronal connectivity as detected by functional MRI. Green arrows indicate radiological correlates of histopathological findings.

Additionally, while CSF biomarkers offer high sensitivity, their requirement for lumbar puncture limits routine use. The measurement of CNS-derived biomarkers in blood offers a minimally invasive alternative to CSF-based assays [312]. Serum NfL (sNfL) values correlate with radiological and clinical measures of disease worsening, however their specificity remains a challenge given their elevation in other neurological conditions [316,317]. GFAP has emerged as a potential biomarker of progressive MS, with higher levels correlating with brain atrophy and worsening disability independent of relapse-driven inflammation [318]. Cytokine and chemokine profiling, particularly CXCL13 in the CSF, was suggested to help in identifying highly inflammatory MS subtypes [319]. The path to routine clinical application would mean tackling challenges such as variability in detection methods across laboratories and the absence of standardized thresholds for defining pathological levels [312]. Practical considerations in the application of blood-based biomarkers into routine practice are the need for assay standardization, high costs of ultra-sensitive detection platforms (e.g. SIMOA, mass spectrometry), and the establishment of clinically meaningful cutoffs that account for individual patient variability [312]. The development of composite panels/scoring systems that integrate markers reflecting different aspects of disease pathology could likely enable more precise disease characterization and treatment monitoring [320]. An example is the ‘Glia Score,’ combining CHI3L1, GFAP, and NfL, proposed as an indicator of neurodegenerative burden in progressive MS [320]. Their implementation in clinical settings would likely need prospective validation in large, real-world MS cohorts, and harmonization of inter-assay variability that limits reproducibility across different research centers [312].

The interpretation of fluid biomarkers is complex and needs correlation with MRI measures or with advanced imaging modalities. Indeed, MRI biomarkers are central to evaluating neuroprotection and repair in MS, yet there remain critical gaps in their ability to accurately capture these processes [321]. Whole-brain and regional atrophy measures have been widely used as endpoints in clinical trials, but likely they primarily reflect irreversible tissue loss rather than ongoing neuroprotective or reparative mechanisms [321]. Emerging MRI techniques, including magnetization transfer ratio (MTR), diffusion tensor imaging (DTI), and myelin water imaging (MWI), provide improved specificity for detecting myelin integrity and axonal damage. MTR and MWI have demonstrated sensitivity to remyelination dynamics in acute white matter lesions, while advanced diffusion-weighted imaging techniques, such as neurite orientation dispersion and density imaging (NODDI), offer insights into axonal integrity [321]. There are, however, practical limitations which hinder their widespread use. Many advanced MRI modalities lack standardization across different MRI platforms, and variations in acquisition protocols, field strength, and post-processing pipelines can lead to significant discrepancies in reported findings [321]. An important point is how the sensitivity of these techniques reflects subtle reparative processes, raising concerns about their utility as primary endpoints in clinical trials [321].

Regarding monitoring of MS progression, aside the change brain volume which is used in clinical trials [68], the imaging of chronic white matter lesions and cortical pathology has gained interest. Susceptibility-weighted imaging (SWI) and quantitative susceptibility mapping (QSM) enable the assessment of iron accumulation and chronic active lesions, with paramagnetic rim lesions serving as potential markers of ongoing neuroinflammation [321]. Cortical lesion detection remains challenging due to the relatively low myelin content in the cortex [322], but advancements in ultra-high-field 7T MRI and phase-sensitive inversion recovery (PSIR) imaging have improved lesion visualization [321], and MRI-based measures of cortical myelin integrity T1/T2 mapping and MTR are being explored as potential tools for tracking cortical demyelination and repair [321]. Distinguishing true cortical lesions from partial volume effects and other artifacts requires highly optimized imaging sequences, which are not universally implemented across MS centers [321]. Lack of standardized imaging protocols for SWI and QSM make cross-site comparisons difficult and 7T MRI remains largely unavailable outside of research settings [321]. Additionally, the slow evolution of chronic lesions complicates the interpretation of longitudinal MRI data, making it difficult to establish clear biomarkers of reparative processes [321]. Addressing these limitations will be crucial to refining MRI biomarkers aiming translation into routine practice, as well as their correlation with the fluid biomarkers to monitor neurodegeneration and outcomes of interventions for MS progression.

It is likely the future of MRI biomarker research in MS lies in multimodal approaches that integrate complementary imaging modalities to improve the assessment of neuroprotection and repair. For example, translocator protein (TSPO) PET imaging provides insight into microglial activation and chronic inflammation and shows extended neuroinflammation in the white matter affected by the paramagnetic rim lesions (PRLs) identified by MRI [323]. PET imaging with myelin-specific tracers, such as 11C-MeDAS, offers direct visualization of myelin repair [324]. Optical coherence tomography (OCT) has the ability to quantify axonal loss in the retina, which correlates with global neurodegenerative changes in MS [325]. Interestingly, OCT and OCT angiography (OCTA) show progressive retinal neurodegeneration and microvascular dysfunction in patients with RRMS without a history of optic neuritis, which strongly correlates with clinical disability [326]. Currently, PET imaging remains generally prohibitively expensive and limited by the availability of specific radiotracers, while OCT requires further validation before being widely used as a surrogate marker for MS progression.

## 9. Expert opinion

Despite the major progress in disease modifying treatment in MS, a definitive solution which can fully salvage axons and repair damage in MS has not been found. As the in-situ maladaptive cascade of chronic CNS inflammation is triggered and amplified by peripheral-driven inflammation, there is no doubt that treating MS early is a way to provide neuroprotection, and that early treatment slows disability accumulation over the long term. The use of high-efficacy DMT early in the disease is associated with better disability outcomes, although this needs to be balanced against side effects [327].

Although initially suppressing peripherally driven inflammation of the CNS with DMT can prevent microstructural tissue damage [50], a major limitation of the currently approved DMT is their failure in tackling over the long term the chronic parenchymal (lesional and extra-lesional) and leptomeningeal inflammation. These are the consequences of the initial focal inflammatory events and are driving neuronal damage and MS progression [4]. A challenge is how to measure/visualize these processes appropriately, in order to gauge the effect of an intervention. As mentioned above, chronic neuroinflammation may prevent repair, and accurately characterizing its extent before a neuroprotective intervention is implemented, and monitoring it during that intervention would be required. MRI can detect the ‘mixed active and inactive lesions’ [328], in which the acute focal inflammation becomes chronic with involvement of monocytes, CD8 memory cells, astrocytes and microglia, this eventually leading to chronic tissue damage. Some of these show the paramagnetic rim sign (iron-laden macrophages bordering the lesion) which is used as a new outcome measure in clinical trials. Visualizing the leptomeningeal tertiary lymphoid structures and inflammation is challenging, but it would be critical as these correlate with subpial demyelination and cortical atrophy and are relevant to progressive MS [329]. Accurately delineating the diffuse microglial activation and multifocal microglial nodules in the ‘normal appearing’ white matter is still an unmet need, although progress was made in positron emission tomography techniques with radioligands that bind to activated microglia and astrocytes [330].

MS pathobiology is dynamic. Disability progression is the consequence of a combination of neuronal injury mechanisms (mitochondrial dysfunction, chronic inflammation and oxidative stress) and failure of repair and buffer mechanisms and of remyelination. A recent extensive review of neurodegeneration in MS, Parkinson’s disease and Alzheimer disease suggests that neurodegeneration should be considered only in triangulation with neuroinflammation and demyelination [18]. A major challenge, which constrains drug development in MS, is how to quantify, in a given individual with MS and at a given time, the contribution of mechanisms of neuronal injury, so to propose an appropriate neuroprotective strategy. Using spatial transcriptomics and proteomics on fresh-frozen human MS brain tissue form people with progressive MS, Kaufmann et al. [331] tracked more than 4,000 paired genes and proteins to identify biological processes expected to occur in the early stages of neurodegeneration [331]. Their results suggest that current drug targets fail to be effective either through their restriction to specific cell types, or due to their inability to impact the pathways identified as relevant [331], which were characterized by multiple levels of redundancy and pertained to a local failure of trophic and anti-inflammatory cellular communication in the early stages of neurodegeneration [331]. How to identify and monitor these to obtain a radiograph of pathological mechanisms at play at a specific time at the single patient level, and to devise relevant measures for it to be translated in trials, is a challenge ahead. The lag between the neuroprotective intervention, and its effects, should be considered accordingly and specifically for each mechanistic intervention, and this delay in showing effects needs to be considered appropriately in clinical trial design, especially in progressive MS [332].

As mentioned above, strategies against mechanisms of neuronal damage in MS (e.g. antioxidants to combat oxidative stress, anti-glutamatergic drugs for excitotoxicity, channel modulators for redistribution of ion channels, cannabinoids for wallerian degeneration, pioglitazone for mitochondrial dysfunction, etc.) do exist, but their translation in practice is complex, as shown by the results of clinical trials [134,333]. The translation from preclinical models to clinical trials is skewed toward antibody and small molecule-based strategies (due to the easier administration) over other types of interventions [19]. While understanding better the cascade of neural injury mechanisms and failure of repair is mandatory, at the same time novel trials should be designed to test neuroprotective interventions. From the clinician’s perspective, a move away from the dogma of classical outcome measures of MS trial is needed, to allow novel strategies for neuroprotection in MS. A specific challenge is posed by the trials in progressive MS [334]. Any neuroprotective strategy in MS should consider that pro-neuroprotective and the neuro-degenerative mechanisms do coexist, with a different ratio and predominance at different MS stages and in different individuals, and are age-, region- and sex-specific. This is true for the remyelination in gray and white mater which coexists with demyelination, and the dual intrinsic neuronal activity with effects on OPC and on resident cells of innate immunity. This complexity makes interpretation of the results of clinical trials with neuroprotective agents challenging. An interesting example is the outcome of the MS-STAT trials. In the phase 2b MS-STAT1 trial [335] which included 140 people with SPMS, simvastatin reduced significantly the rate of brain atrophy vs placebo. In the phase 3 MS-STAT2 trial on 964 people with SPMS, simvastatin was not effective in slowing disability progression [336]. While further analysis on other measures such as atrophy is pending, it is not clear if the discrepancy between the trial results is due to regression to the mean, or to other factors pertaining to the populations included. Interestingly, while statins can have immunomodulatory effects which are beneficial in MS, and possibly can be neuroprotective [337], they can also inhibit remyelination in animal models [338].

Neuroprotective strategies can focus on one target, or multiple targets. Whilst the first approach provides more mechanistic clarity, and it is easier to monitor in terms of target outcome engagement and side effects, its effects may not be always clinically meaningful in a complex disease context, where mechanisms leading to neuronal damage intertangle with failure of compensatory mechanisms.

A multi-target approach (BTK-inhibitors are a representative example) is likely to be more successful, however interpreting the measures of the effects of such an intervention has its challenges and requires moving away from the classical trial paradigm of measures consisting in number of MRI lesions and clinical relapses, and reappraise the biological interpretation of radiological measures such atrophy or enhancing lesions. In the phase 3 GEMINI trials in relapsing MS, although there was no significant difference in relapse rate between tolebrutinib and teriflunomide, and although the number of new Gd-enhancing T1 lesions was higher in the tolebrutinib arm, tolebrutinib showed a 29% risk reduction in 6-month clinical disability worsening vs. teriflunomide [57]. In the phase 3 hERCULES trial in people with non-relapsing secondary progressive MS, tolebrutinib was associated with a 31% risk reduction in time to 6-month confirmed disability progression vs. placebo, and significantly lowered the annualized rate of new/enlarging T2 lesions, but the brain volume loss difference was not significant [339]. Whilst these results suggest that acute focal MS inflammation and smoldering neuroinflammation are two distinct biological processes, they also suggest the need for novel measures for correlative assessment of clinical effects of neuroprotection: measures of axonal viability, of CNS neuroinflammatory load/spread, and of amount of remyelination; and exploration of how these correlate with biological biomarkers such as NfL and GFAP. Design of MS trials could be informed by measures and outcomes of brain target engagement for neuroprotection from other diseases driven by neurodegeneration, such as Parkinson’s disease [340].

Although microglia are rightly considered central to smoldering MS, recent methodological progress in studying brain resident cells in vivo shows astrocytes being crucial to MS progression. Quintana et al. combined barcoded viral tracing and single-cell RNA sequencing to create RABID-seq (rabies barcode interaction detection followed by sequencing) which further allowed them to identify and study microglia-astrocyte interactions [341]. They showed that axon guidance molecules Sema4DPlexinB1, Sema4D-PlexinB2, and Ephrin-B3/EphB3 are mediators of microglia-astrocyte interactions in EAE and potential candidate targets for treatment in MS [341]. Astrocyte epigenetic memory enhances pro-inflammatory responses and promotes CNS pathology in EAE and MS [342]. Activated astrocytes are part of smoldering MS lesions [343], neurotoxic reactive astrocytes can be induced by activated microglia [344], and the axis between activated inflammatory microglia and activated inflammatory astrocytes is central to the glial interactome in chronic active MS [343], where astrocytes interacted also with stressed oligodendroglia, thus influencing remyelination [343]. Recently, the C-type lectin domain-containing 16A gene, linked to MS susceptibility, was identified as a suppressor of astrocyte pathological responses and a candidate therapeutic target in MS [345]. It is the authors’ prediction that a new arm of therapeutics for MS targeting specifically astrocytes will likely be developed in the years to come, as new potential tools to tackle MS progression. As prognostication of future disability in MS can be made based on a single GFAP measurement from serum, serum GFAP could be used in trial as progression biomarker for this pipeline of drug development [318].

The future of strategies for neuroprotection includes also the design of drugs which can target macrophage/microglia functioning, microglia-astrocyte crosstalk, and astrocytic modulation [346]. This includes pipelines of screening and drug repurposing relevant to single-cell interactions and phenotypes in the CNS [341,347]. Harnessing the axonal response of mitochondria to demyelination (ARMD) can be a pre-requisite step for applying a potential neuroprotective/neurodegenerative strategy in MS [348]. The candidates from preclinical studies can be trialed in multi-arm multistage adaptive trials (MAMSAT), which have the advantage of a more rapid and adapted evaluation of new drugs, in comparison with traditional clinical trials [349]. MAMSAT can address multiple research questions simultaneously under a single trial protocol and regulatory framework and they efficiently allow adding new arms while the study is ongoing [350]. This means MAMSAT possibly can decrease the number of patients treated with ineffective medications or at ineffective doses and maximize the numbers treated on more efficacious medications, thus being more beneficial for the enrolled patients [350]. The main limitations of MAMSAT consist in being more resource intensive due to the multiple arms and multiple stages, especially in the initial design phases [350]. The increased complexity of MAMSAT involves greater specialist statistical input and makes them potentially less attractive to investigators not used with this design [351,352]. A main risk is down to the smaller number of subjects in the interim analyses which means potentially effective medications could be excluded if they fulfill the predefined stopping rules [351].

Finally, when to apply the neuroprotection interventions in MS is a crucial point in their success. Trying effective neuroprotection in advanced progressive MS is probably to no avail, as neuronal damage has already ensured. Envisaging neuroprotective effects of drugs in early/very early MS means also developing adequate measures for those effects [353], and having a combined/multiple biomarkers for monitoring.

Probably the greatest progress in planning neuroprotection will be brought by better understanding of the very early changes occurring in the CNS relevant to neurodegeneration in MS, which should be mitigated as early as possible so to salvage neurons and restore CNS homeostasis to a pro-neuroprotective environment.

MS represents a disease continuum in which inflammation and neurodegeneration co-occur from onset and evolve over time [354]. The recent progress in the understating of MS creates new perspectives for novel neuroprotective therapeutic strategies [354]. Adequate biomarkers to help define, monitor, or predict MS course at the individual level are needed. Novel measures and target outcomes are needed to measure the effect of neuroprotective interventions, in multi-arm, multistage adaptive trials. Strategies of neuroprotection in MS should take advantage from translation of cross-disease paradigms relevant across multiple neurodegenerative diseases. The outcome of neuroprotective strategies is likely to be positive only if they are implemented as early as possible, before the maladaptive microglial and astrocytic modifications establish themselves in the CNS, and certainly before significant neuronal loss has occurred. Effects of aging, comorbidities and lifestyle factor modification should be considered, both as factors influencing the pathobiology of MS relevant to neurodegeneration, and as targets for neuroprotective interventions.

## Supplementary Material

Supplemental Material

## Article highlights

- Demyelination, myelin/oligodendroglial loss and axonal pathology are the main contributors to neurodegeneration.
- Molecular pathways involved in axonal damage and neurodegeneration include oxidative stress, calcium overflow, excitotoxicity and mitochondrial damage. Strategies that aim to promote neuroprotection should be able to prevent, reverse and repair the damage produced along these pathways.
- There is a need for novel measures and correlative assessment of clinical effects of neuroprotection, such as measures of axonal viability, of CNS neuroinflammation, and of the amount of remyelination.
- The design of MS trials could be informed by measures and outcomes of brain target engagement for neuroprotection from other diseases driven by neurodegeneration.
- Targeting the maladaptive functional changes of CNS resident cells – macrophage/microglia and astrocytes, and addressing the axonal response of mitochondria to demyelination (ARMD) are strategies for neuroprotection.
- Candidate neuroprotective drugs from preclinical studies and early clinical studies can be tested in multi-arm multistage adaptive trials. Further research is required to address the translational challenges of preclinical models and neuroprotective interventions, to people with MS
