# Supplementary material for: Neuroprotective strategies in multiple sclerosis: a status update and emerging paradigms
Source: Expert Rev Neurother. 2025 Jun 3;25(7):791–817. doi: 10.1080/14737175.2025.2510405 (PMC12312756; doi:10.1080/14737175.2025.2510405)
Supplement: Supplemental Material [file IERN_A_2510405_SM0252.docx]

Supplementary Table 1 – Examples of neuroprotective strategies.

| **Neuroprotective target** | **Molecule / Strategy** | **Methods**  **Culture (C ), animal (A), Human (H)** |
| --- | --- | --- |
| **Oxidative stress** | Alpha-lipoic acid (LA) - natural anti-oxidant, blocks free radicals. | A (EAE - experimental autoimmune encephalitis) [1] |
|  | enhancing microglia-mediated OxPC (oxidized phosphatidylcholines) clearance via TREM2 (trigger receptor expressed on myeloid cells 2) | A(EAE) [2] |
|  | inhibiting NF-κB and by inducing Nrf2/Keap1 | A [4] |
|  | Hesperidin: ↓ MDA (malondialdehyde), ↑ GPx (glutathion peroxidase), ↑ SOD(superoxide dismutase), CAT (catalase), ↑ GSH (glutathione) | A [5] (EAE) |
| **Mitochondrial dysfunction** | JW47: cyclophilin D (CyPD) inhibitor and permeability transition pore (PTP) as potential targets in neuroprotection | A (EAE) [6] |
|  | Rapamycin, everolimus: mTOR inhibitors, mitochondriogenic effects | A (non-obese diabetic NOD mouse model of progressive MS) [7] |
|  | Mitochondrial transplantation | A (EAE) [8,9] |
|  | Promoting ARMD ( axonal response of mitochondria to demyelination) neuroprotective strategy for the vulnerable, acutely demyelinated axon | A [10] |
| **Ion dysregulation** | Inhibition of transient receptor potential melastatin 2 (TRPM2) channel- Ca 2+ mediated inducing microglial cell activation | A (EAE) [11] |
|  | Nimodipine: L type voltage-gated calcium channel (VGCC) blocker | A (EAE). [12] |
| **Glutamate mediated excitotoxicity** | ZCAN262: AMPAR (alpha-amino-3-hydroxy-5-methyl-4-isooxazole-propionic acid receptor)- GluA2 modulator | A (EAE) [13] |
|  | deletion of AMPAR subunit GluA4 on mature oligodendrocytes | A (EAE) [14] |
|  | TG-2112x : sequestration of mitochondrial calcium uptake | C [15] |
| **Epigenetic mechanisms** | Epigenetic modification of forkhead box P (FOXP3) gene | A (EAE) [16] |
|  | Vorinostat: histone deacetylase inhibitors (HDACi) | A (EAE) [17] |
|  | Trichostatin A: histone hyperacetylation reduced inflammation | A (NOD mice) [18] |
| **Neuronal stress response and immune signaling** | Decreasing autoimmunity via astrocytes IFN (interferon) γ signaling during chronic autoimmunity via upregulation of PD-L1 (programmed death ligand 1) | A (EAE) [19] |
|  | targeting neuronal stimulator of interferon genes (STING) genetically and pharmacologically | A, C [20] |
|  | STING1 inhibitors: neuron-specific deletion of Sting 1 | A (EAE)[21] |
| **Unfolded protein response** | activation of pancreatic endoplasmic reticulum kinase (PERK) | A (EAE)[22] |
| **Protein accumulation** | Pharmacological enhancement of proteasomal activity increases Bassoon protein (BSN) clearance | A [23] |
| **Mechanisms of neuronal cell death -ferroptosis, necroptosis** | liproxstatin-1: inhibiting ferroptosis | A (EAE) [24] |
|  | Decreasing ACSL4 (acyl-CoA synthetase long-chain family member 4) | A (EAE)[24] |
|  | UAMC-3203: inhibiting ferroptosis | A (EAE) [25] |
|  | SAR443820 (DNL788): inhibitor of Receptor-interacting serine/threonine-protein kinase 1 (RIPK1); inhibiting necroptosis. | A, H [26] |
| **Remyelination** | Clemastine: muscarinic antagonist, promoting oligodendrogenesis. | H [27] |
|  | Bazedoxifene: selective estrogen modulator; promoting remyelination potentially through modulating p38 and ERK pathways. | A (EAE) [28] |
|  | ZJU37: RIPK1 inhibitor promoting oligodendrocyte proliferation | A [29] |
|  | Miconazole, clobetasole: enhance oligodendrocyte generation; act on oligodendrocyte progenitor cells through mitogen-activated protein kinase and glucocorticoid receptor signaling, | A [30] |
|  | Ultrasound neuromodulation: accelerating remyelination through the attenuation of glial activation and the enhancement of mature oligodendrocyte density and BDNF production. | A (lysolecithin) [31]l |

REFERENCES

1. Xie H, Yang X, Cao Y, Long X, Shang H, Jia Z. Role of lipoic acid in multiple sclerosis. CNS Neurosci Ther. 2022 Mar;28(3):319–31.

2. Dong Y, D’Mello C, Pinsky W, Lozinski BM, Kaushik DK, Ghorbani S, Moezzi D, Brown D, Melo FC, Zandee S, Vo T, Prat A, Whitehead SN, Yong VW. Oxidized phosphatidylcholines found in multiple sclerosis lesions mediate neurodegeneration and are neutralized by microglia. Nat Neurosci. 2021 Apr;24(4):489–503.

3. Santos M, Maurício T, Domingues R, Domingues P. Impact of oxidized phosphatidylcholine supplementation on the lipidome of RAW264.7 macrophages. Arch Biochem Biophys. 2025 Jun;768:110384.

4. Buendia I, Michalska P, Navarro E, Gameiro I, Egea J, León R. Nrf2–ARE pathway: An emerging target against oxidative stress and neuroinflammation in neurodegenerative diseases. Pharmacol Ther. 2016 Jan;157:84–104.

5. Zha Z, Liu S, Liu Y, Li C, Wang L. Potential Utility of Natural Products against Oxidative Stress in Animal Models of Multiple Sclerosis. Antioxid Basel Switz. 2022 Jul 29;11(8):1495.

6. Warne J, Pryce G, Hill JM, Shi X, Lennerås F, Puentes F, Kip M, Hilditch L, Walker P, Simone MI, Chan AWEdit, Towers GJ, Coker AR, Duchen MR, Szabadkai G, Baker D, Selwood DL. Selective Inhibition of the Mitochondrial Permeability Transition Pore Protects against Neurodegeneration in Experimental Multiple Sclerosis. J Biol Chem. 2016 Feb;291(9):4356–73.

7. Buonvicino D, Pratesi S, Ranieri G, Pistolesi A, Guasti D, Chiarugi A. The mitochondriogenic but not the immunosuppressant effects of mTOR inhibitors prompt neuroprotection and delay disease evolution in a mouse model of progressive multiple sclerosis. Neurobiol Dis. 2024 Feb;191:106387.

8. Peruzzotti-Jametti L, Bernstock JD, Willis CM, Manferrari G, Rogall R, Fernandez-Vizarra E, Williamson JC, Braga A, van den Bosch A, Leonardi T, Krzak G, Kittel Á, Benincá C, Vicario N, Tan S, Bastos C, Bicci I, Iraci N, Smith JA, Peacock B, Muller KH, Lehner PJ, Buzas EI, Faria N, Zeviani M, Frezza C, Brisson A, Matheson NJ, Viscomi C, Pluchino S. Neural stem cells traffic functional mitochondria via extracellular vesicles. PLoS Biol. 2021 Apr;19(4):e3001166.

9. Picone P, Nuzzo D. Promising Treatment for Multiple Sclerosis: Mitochondrial Transplantation. Int J Mol Sci. 2022 Feb 17;23(4):2245.

10. Licht-Mayer S, Campbell GR, Canizares M, Mehta AR, Gane AB, McGill K, Ghosh A, Fullerton A, Menezes N, Dean J, Dunham J, Al-Azki S, Pryce G, Zandee S, Zhao C, Kipp M, Smith KJ, Baker D, Altmann D, Anderton SM, Kap YS, Laman JD, Hart BA ‘t, Rodriguez M, Watzlawick R, Schwab JM, Carter R, Morton N, Zagnoni M, Franklin RJM, Mitchell R, Fleetwood-Walker S, Lyons DA, Chandran S, Lassmann H, Trapp BD, Mahad DJ. Enhanced axonal response of mitochondria to demyelination offers neuroprotection: implications for multiple sclerosis. Acta Neuropathol (Berl). 2020 Aug;140(2):143–67.

11. Tsutsui M, Hirase R, Miyamura S, Nagayasu K, Nakagawa T, Mori Y, Shirakawa H, Kaneko S. TRPM2 Exacerbates Central Nervous System Inflammation in Experimental Autoimmune Encephalomyelitis by Increasing Production of CXCL2 Chemokines. J Neurosci. 2018 Sep 26;38(39):8484–95.

12. Schampel A, Volovitch O, Koeniger T, Scholz CJ, Jörg S, Linker RA, Wischmeyer E, Wunsch M, Hell JW, Ergün S, Kuerten S. Nimodipine fosters remyelination in a mouse model of multiple sclerosis and induces microglia-specific apoptosis. Proc Natl Acad Sci [Internet]. 2017 Apr 18 [cited 2025 Mar 24];114(16). Available from: https://pnas.org/doi/full/10.1073/pnas.1620052114

13. Wood H. GluA2 modulator targets excitotoxicity in MS. Nat Rev Neurol. 2024 Feb;20(2):63–63.

14. Evonuk KS, Doyle RE, Moseley CE, Thornell IM, Adler K, Bingaman AM, Bevensee MO, Weaver CT, Min B, DeSilva TM. Reduction of AMPA receptor activity on mature oligodendrocytes attenuates loss of myelinated axons in autoimmune neuroinflammation. Sci Adv. 2020 Jan;6(2):eaax5936.

15. Angelova PR, Vinogradova D, Neganova ME, Serkova TP, Sokolov VV, Bachurin SO, Shevtsova EF, Abramov AY. Pharmacological Sequestration of Mitochondrial Calcium Uptake Protects Neurons Against Glutamate Excitotoxicity. Mol Neurobiol. 2019 Mar;56(3):2244–55.

16. Noori-Zadeh A, Mesbah-Namin SA, Saboor-Yaraghi AA. Epigenetic and gene expression alterations of FOXP3 in the T cells of EAE mouse model of multiple sclerosis. J Neurol Sci. 2017 Apr;375:203–8.

17. Ge Z, Da Y, Xue Z, Zhang K, Zhuang H, Peng M, Li Y, Li W, Simard A, Hao J, Yao Z, Zhang R. Vorinostat, a histone deacetylase inhibitor, suppresses dendritic cell function and ameliorates experimental autoimmune encephalomyelitis. Exp Neurol. 2013 Mar;241:56–66.

18. Jayaraman A, Soni A, Prabhakar BS, Holterman M, Jayaraman S. The epigenetic drug Trichostatin A ameliorates experimental autoimmune encephalomyelitis via T cell tolerance induction and impaired influx of T cells into the spinal cord. Neurobiol Dis. 2017 Dec;108:1–12.

19. Smith BC, Tinkey RA, Brock OD, Mariam A, Habean ML, Dutta R, Williams JL. Astrocyte interferon-gamma signaling dampens inflammation during chronic central nervous system autoimmunity via PD-L1. J Neuroinflammation. 2023 Oct 12;20(1):234.

20. Woo MS, Mayer C, Binkle-Ladisch L, Sonner JK, Rosenkranz SC, Shaposhnykov A, Rothammer N, Tsvilovskyy V, Lorenz SM, Raich L, Bal LC, Vieira V, Wagner I, Bauer S, Glatzel M, Conrad M, Merkler D, Freichel M, Friese MA. STING orchestrates the neuronal inflammatory stress response in multiple sclerosis. Cell. 2024 Jul 25;187(15):4043-4060.e30.

21. Houston S. Putting the STING in MS. Nat Immunol. 2024 Aug;25(8):1309–1309.

22. Stone S, Yue Y, Stanojlovic M, Wu S, Karsenty G, Lin W. Neuron-specific PERK inactivation exacerbates neurodegeneration during experimental autoimmune encephalomyelitis. JCI Insight. 2019 Jan 24;4(2):e124232.

23. Montenegro-Venegas C, Fienko S, Anni D, Pina-Fernández E, Frischknecht R, Fejtova A. Bassoon inhibits proteasome activity via interaction with PSMB4. Cell Mol Life Sci. 2021 Feb;78(4):1545–63.

24. Luoqian J, Yang W, Ding X, Tuo Q zhang, Xiang Z, Zheng Z, Guo Y jie, Li L, Guan P, Ayton S, Dong B, Zhang H, Hu H, Lei P. Ferroptosis promotes T-cell activation-induced neurodegeneration in multiple sclerosis. Cell Mol Immunol. 2022 Jun 8;19(8):913–24.

25. Van San E, Debruyne AC, Veeckmans G, Tyurina YY, Tyurin VA, Zheng H, Choi SM, Augustyns K, Van Loo G, Michalke B, Venkataramani V, Toyokuni S, Bayir H, Vandenabeele P, Hassannia B, Vanden Berghe T. Ferroptosis contributes to multiple sclerosis and its pharmacological targeting suppresses experimental disease progression. Cell Death Differ. 2023 Sep;30(9):2092–103.

26. Xavier Montalban. Effect of RIPK1 Inhibitor, SAR443820, on Serum Neurofilament Light Levels in Patients with Multiple Sclerosis: A Phase 2 Trial Design. AAN P6 Abstr 53458.

27. Green AJ, Gelfand JM, Cree BA, Bevan C, Boscardin WJ, Mei F, Inman J, Arnow S, Devereux M, Abounasr A, Nobuta H, Zhu A, Friessen M, Gerona R, Von Büdingen HC, Henry RG, Hauser SL, Chan JR. Clemastine fumarate as a remyelinating therapy for multiple sclerosis (ReBUILD): a randomised, controlled, double-blind, crossover trial. The Lancet. 2017 Dec;390(10111):2481–9.

28. Rankin KA, Mei F, Kim K, Shen YAA, Mayoral SR, Desponts C, Lorrain DS, Green AJ, Baranzini SE, Chan JR, Bove R. Selective Estrogen Receptor Modulators Enhance CNS Remyelination Independent of Estrogen Receptors. J Neurosci. 2019 Mar 20;39(12):2184–94.

29. Ma XR, Yang SY, Zheng SS, Yan HH, Gu HM, Wang F, Wu Y, Dong ZJ, Wang DX, Wang Y, Meng X, Sun J, Xia HG, Zhao JW. Inhibition of RIPK1 by ZJU-37 promotes oligodendrocyte progenitor proliferation and remyelination via NF-κB pathway. Cell Death Discov. 2022 Apr 1;8(1):147.

30. Najm, F., Madhavan, M., Zaremba, A. *et al.* Drug-based modulation of endogenous stem cells promotes functional remyelination *in vivo*. *Nature* **522**, 216–220 (2015).

31. Yang FY, Huang LH, Wu MT, Pan ZY. Ultrasound Neuromodulation Reduces Demyelination in a Rat Model of Multiple Sclerosis. Int J Mol Sci. 2022 Sep 2;23(17):10034.
